# Clinical risk scores for stroke correlate with molecular signatures of vulnerability in symptomatic carotid patients

**DOI:** 10.1016/j.isci.2022.104219

**Published:** 2022-04-08

**Authors:** Katarina Wadén, Eva Karlöf, Sampath Narayanan, Mariette Lengquist, Göran K. Hansson, Ulf Hedin, Joy Roy, Ljubica Matic

**Affiliations:** 1 Vascular Surgery, Department of Molecular Medicine and Surgery, Karolinska Institutet and Karolinska University Hospital, 17176 Stockholm, Sweden; 2 Cardiovascular Medicine Unit, Department of Medicine, Center for Molecular Medicine, Karolinska Institutet and Karolinska University Hospital, 17176 Stockholm, Sweden

**Keywords:** Health sciences, Transcriptomics, Cell biology

## Abstract

Unstable carotid stenosis is an important cause of ischemic stroke, yet the basis of disease pathophysiology remains largely unknown. We hypothesized that integrated analyses of symptomatic carotid stenosis patients at increased stroke risk stratified by clinical scores, CAR and ABCD2, with transcriptomic and clinical data could improve identification of molecular pathways and targets for instability. We show that high CAR score reflects plaque instability processes related to intra-plaque hemorrhage, angiogenesis, inflammation, and foam cell differentiation, whereas ABCD2 associates with neutrophil-mediated immunity, foam cell differentiation, cholesterol transport, and coagulation. Repressed processes in plaques from high-risk patients were ossification, chondrocyte differentiation, SMC migration, and ECM organization. *ABCB5* gene was found as the top upregulated in high-risk patient’s plaques, localized to macrophages in areas with neovascularization and intra-plaque hemorrhage. The link between *ABCB5* and intra-plaque hemorrhage suggests its key role for plaque instability that warrants further exploration.

## Highlights

• We integrated stroke risk in carotid stenosis patients with plaque transcriptomics
• High CAR and ABCD2 scores reflect plaque instability processes and hemorrhage
• *ABCB5* is upregulated in high-risk plaques, macrophages, and around neovessels
• CAR and ABCD2 capture vulnerable plaque features and improve risk stratification

## Graphical abstract

**Figure:**
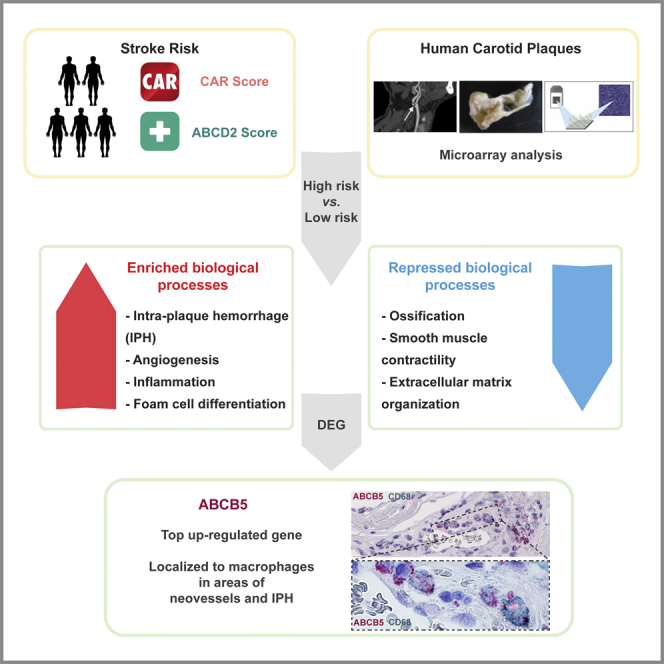

## Introduction

Stroke is one of the major causes of death and disability from cardiovascular disease (CVD) (World Health Organization, 2011), and thromboembolism from carotid stenosis (CS) is the cause of ischemic strokes (IS) in 10-20% cases (Cheng et al., 2019; Naylor, 2015). Smoking, hypertension, diabetes, advancing age, male sex, hyperlipidemia, coronary- and peripheral vascular disease, genetic predisposition, and physical inactivity are known risk factors for atherosclerotic plaque development, CS, and IS (Folkersen et al., 2012; Mughal et al., 2011). Effective prevention of ischemic strokes demands identification of individuals and lesions at high-risk, but most utilized clinical predictors are still based on surrogates without firm associations to the underlying disease pathophysiology, atherosclerotic plaque instability. Despite the availability of various imaging modalities, plasma biomarkers, and risk scoring systems, patients are usually identified after, rather than before symptoms.

The pathophysiology behind carotid plaque instability is complex and involves activation of inflammatory processes, formation of a lipid-rich necrotic core including cell apoptosis, protease activity with extracellular matrix (ECM) degradation causing the thinning of the fibrous cap, altogether leading to thrombogenicity and platelet activation (Hansson et al., 2015). Neovascularization induced by hypoxia has been described as a strong contributor to plaque progression, instability, and rupture because of immature and leaky neovessels, which eventually cause intra-plaque hemorrhage (IPH) (Parma et al., 2017).

To understand how patient phenotype corresponds to carotid plaque instability and identify new molecular targets for diagnostic or therapeutic approaches, we have previously correlated different clinical parameters to gene expression patterns in endarterectomy specimens from our Biobank of Karolinska Endarterectomies (BiKE). Using this rationale, enrichment of molecular pathways have been determined that distinguish patient phenotypes (i.e., comparing symptomatic patients with asymptomatic ones or effects of statin treatment) but also plaque phenotypes (i.e. calcification or lipid rich necrotic core content) (Perisic et al., 2016; Matic et al., 2018; Karlöf et al., 2021). However, it is unknown if more detailed risk stratification of symptomatic patients in particular could also correspond to biological features representing the severity of plaque instability.

Symptomatic patients show considerable diversity, where further clinical stratification could be achieved by various stroke risk prediction scoring tools. ABCD2 is an easily accessible, elementary, universal prediction tool for short-term risk for stroke (days-to-months), aimed to guide management of patients with a transient ischemic attack (TIA) in primary care or emergency settings (Johnston et al., 2007; Giles and Rothwell, 2010). Carotid Artery Risk (CAR) score is a more detailed, CS specific risk prediction tool that estimates a 5-year ipsilateral IS in recently symptomatic patients on secondary preventive medication (Rothwell and Warlow, 1999; Rothwell et al., 2004; Rothwell et al., 2005; European Carotid Surgery Trial, 2015a).

Here, we hypothesized that the ABCD2 and CAR scores correlate with plaque vulnerability features when applied to further stratify risk particularly in symptomatic patients. Our aim was to investigate if this stratification could determine molecular signatures of plaque instability, using global gene expression profiling of plaques and circulating cells in patients with symptomatic CS. For comparison, ABCD2 and CAR were also associated with patient clinical data and blood parameters.

## Results

### Study workflow and correlation of risk scores with patient clinical data

**Figure 1:**
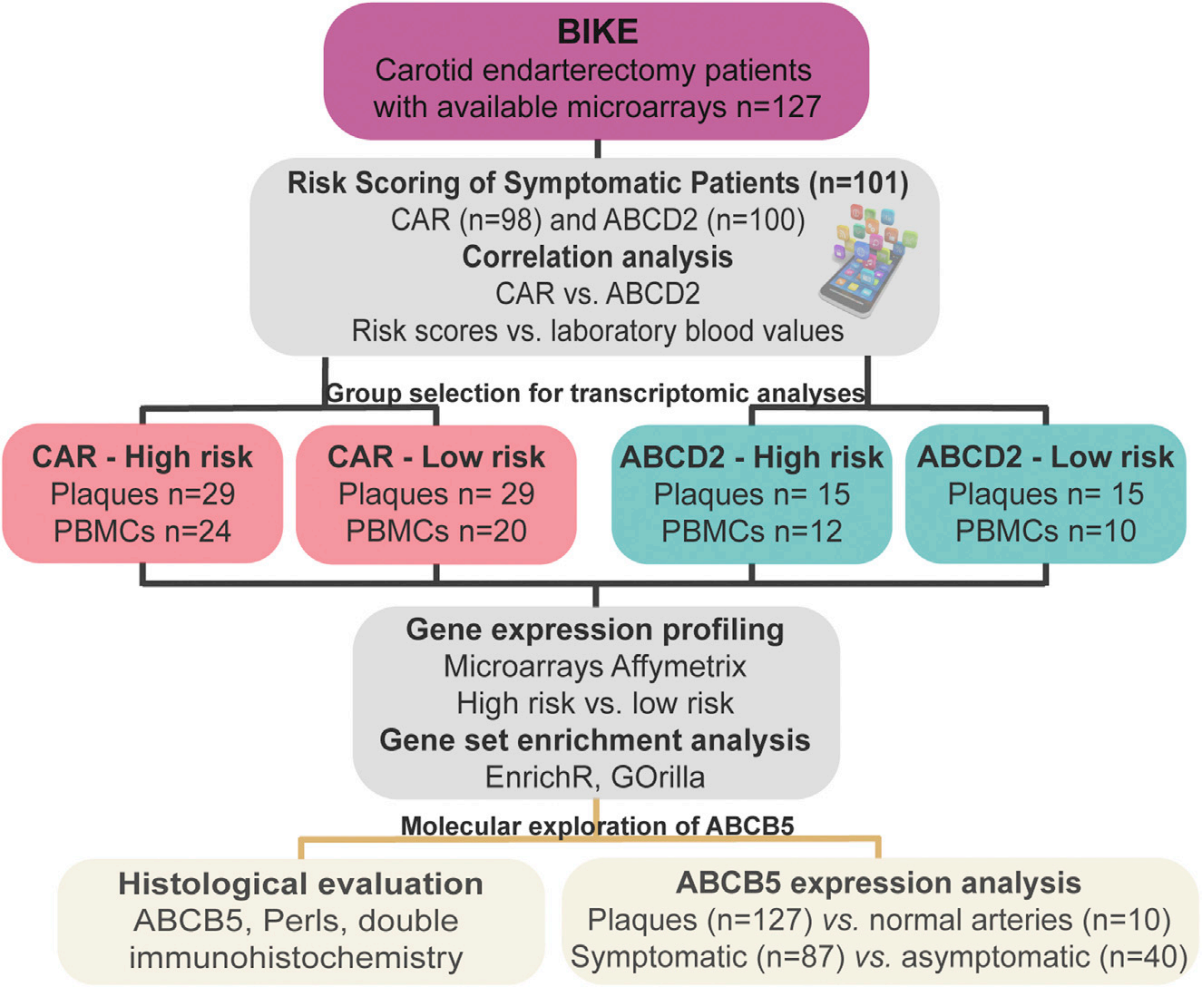
Study workflow and correlation of risk scores with patient clinical data in the Biobank of Karolinska Endarterectomies (BiKE) See also Table S1.Symptomatic CEA patients (n = 101) were scored by CAR (n = 98) and ABCD2 (n = 100) and their calculated risks for stroke were correlated with various blood parameters. Bioinformatic transcriptomic analyses were then performed comparing high-risk vs. low-risk groups in microarrays from plaques and peripheral blood monocytes (PBMCs). Top candidate was validated by immunohistochemistry and gene expression analysis in extended BiKE patients (n = 127) and in normal arteries (n = 10).

A total of n = 101 patients treated with carotid endarterectomy (CEA) were successively enrolled in the study based on symptoms before surgery and eligibility for risk scoring by CAR (n = 98) and ABCD2 (n = 100) (Figure 1, scoring parameters given in Table S1). The risk scores were calculated and analyzed in association to blood parameters. Bioinformatic analysis was performed comparing global transcriptomic profiles of plaques and peripheral blood monocytes (PBMCs) from high vs. low clinical risk score groups, followed by gene set enrichment analysis and pathway mapping. The most significantly dysregulated gene identified through these analyses was investigated further in an extended sub-cohort of BiKE plaques using microarrays and immunohistochemistry.

Overall, the study cohort consisted mainly of male subjects (67%) with a mean age of 72.4 years and a mean body mass index of 26.2 (Table 1). A more detailed demographic analysis showed that the high and low-risk groups in both CAR and ABCD2 scores were comparable with respect to smoking, hypertension, peripheral vascular disease, and major classes of medications. In relation to other comorbidities and symptoms, ocular symptoms were expected to be more prevalent in the low-risk groups, whereas diabetes, TIA, major stroke, and history of myocardial infarction were prevalent in high-risk groups.

**Table 1:** Demographics of patient cohorts

GENERAL	All	CAR^a^			ABCD2		
		Low-risk	High-risk	p-value	Low-risk	High-risk	p-value
Number of patients	101	29	29	na^b^	15	15	na
Age – years	72.29 (±0.83)	71.03 (±1.51)	73.38 (±1.63)	ns^c^	70.00 (±2.54)	73.67 (±2.46)	ns
Sex, women/men	33/68 (33/67)	10/19 (35/65)	5/24 (17/83)	ns	7/8 (47/53)	6/9 (40/60)	ns
Smoking, present	24 (24)	6 (21)	6 (21)	ns	7 (47)	3 (20)	ns
Smoking, former	28 (28)	9 (31)	7 (24)	ns	4 (27)	3 (20)	ns
Smoking, never	49 (49)	14 (48)	17 (59)	ns	4 (27)	9 (60)	ns
BMI^d^	26.2 (±0.43)	26.6 (±0.90)	26.3 (±0.78)	ns	28.1 (±1.07)	26.7 (±1.69)	ns
Hypertension	96(95)	29 (100)	28 (97)	ns	15 (100)	15 (100)	na
Myocardial Infarction	24 (24)	4 (14)	14 (48)	0.0045	6 (40)	5 (33)	ns
Diabetes Mellitus	27 (27)	5 (17)	14 (48)	0.0118	4 (27)	5 (33)	ns
PVD^e^	14 (13)	3 (10)	5 (17)	ns	3 (20)	3 (20)	ns
Antihypertensives	83 (82)	26 (90)	26 (90)	ns	14 (93)	13 (86.7)	ns
Antidiabetics	23 (23)	4 (14)	12 (41)	0.0188	3 (20)	5 (33)	ns
Lipid lowering drugs	87 (86)	27 (93)	25 (86)	ns	13 (87)	13 (87)	ns
Amaurosis fugax	23 (23)	13 (45)	0	<0.0001	11 (73)	0	<0.0001
TIA^f^	37 (37)	9 (31)	11 (38)	ns	4 (27)	15 (100)	<0.0001
Minor stroke	11 (11)	4 (14)	5 (17)	ns	0	0	na
Major stroke	30 (30)	3 (10)	13 (45)	0.0033	0	0	na

Descriptive clinical data for all patients included in this study, as well as for the low and high-risk CAR and ABCD2 groups with available plaque microarrays.

Data are presented as n (%) or mean (±SEM).

a CAR = Carotid Artery Risk score.

b na = not applicable.

c ns = non-significant.

d BMI = Body Mass Index.

e PVD = Peripheral Vascular Disease.

f TIA = Transient Ischemic Attack.

**Figure 2:**
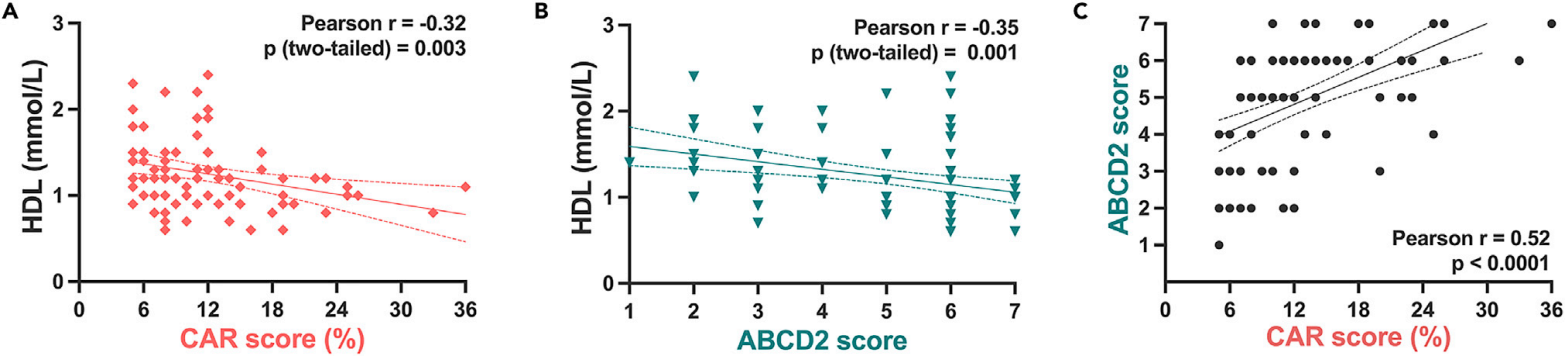
Correlation of risk scores to high-density lipoprotein (HDL) and to each other See also Table 2. (A) CAR was negatively correlated with patient plasma HDL levels. (B) ABCD2 was negatively correlated with patient plasma HDL levels. (C) Clinical risk scores CAR and ABCD2 in carotid patients were moderately significantly correlated with each other.

The association of risk scores with blood parameters (Table 2) showed positive correlation of CAR score to fibrinogen, white blood cell (WBC) count, and s-creatinine (in extension also a significant correlation with estimated Glomerular Filtration rate (eGFR)), whereas an inverse correlation was observed with hemoglobin (Hb) levels. ABCD2 did not correlate with the examined blood parameters, except that both ABCD2 and CAR showed inverse significant correlation to plasma high-density lipoprotein (HDL) levels (Figures 2A and 2B). There was no correlation with CRP for either of the risk scores. The mutual association between CAR and ABCD2 risk scores was also evaluated and found to be moderately positive but strongly significant (r = 0.52, p < 0.0001) (Figure 2C).

**Table 2:** Correlation of clinical risks for stroke estimated by CAR or ABCD2 with patient blood measurements

CAR score vs.	Pearson r	95% confidence interval	P (two-tailed)
Fibrinogen	0.22	0.01699 to 0.4017	0.034
eGFR^a^	−0.33	−0.5066 to −0.1219	0.002
S-Creatinine	0.40	0.2015 to 0.5648	<0.001
S-Cholesterol	−0.14	−0.3273 to 0.06663	0.188
Triglycerides	−0.03	−0.2291 to 0.1716	0.772
Low-density lipoproteins	−0.03	−0.2454 to 0.1883	0.789
High-density lipoproteins	−0.32	−0.4972 to −0.1096	0.003
Hemoglobin	−0.21	−0.3903 to −0.005724	0.044
White blood cell count	0.22	0.02193 to 0.4040	0.030
hs-CRP^b^	0.04	−0.1747 to 0.2509	0.717

**ABCD2 score vs.**			

Fibrinogen	0.15	−0.04660 to 0.3432	0.131
eGFR^a^	−0.18	−0.3814 to 0.02861	0.089
S-Creatinine	0.19	−0.02249 to 0.3866	0.079
S-Cholesterol	−0.05	−0.2487 to 0.1471	0.605
Triglycerides	−0.03	−0.2250 to 0.1716	0.786
Low-density lipoproteins	0.06	−0.1590 to 0.2710	0.598
High-density lipoproteins	−0.35	−0.5224 to −0.1458	0.001
Hemoglobin	−0.16	−0.3472 to 0.03993	0.116
White blood cell count	0.15	−0.05285 to 0.3357	0.148
hs-CRP^b^	−0.07	−0.2797 to 0.1394	0.499

See also Figure 2.

a eGFR = estimated Glomerular Filtration Rate.

b hs-CRP = high-sensitivity C-reactive Protein.

### High CAR score associates with IPH, angiogenesis, foam cell differentiation, and inflammation in plaques

**Figure 3:**
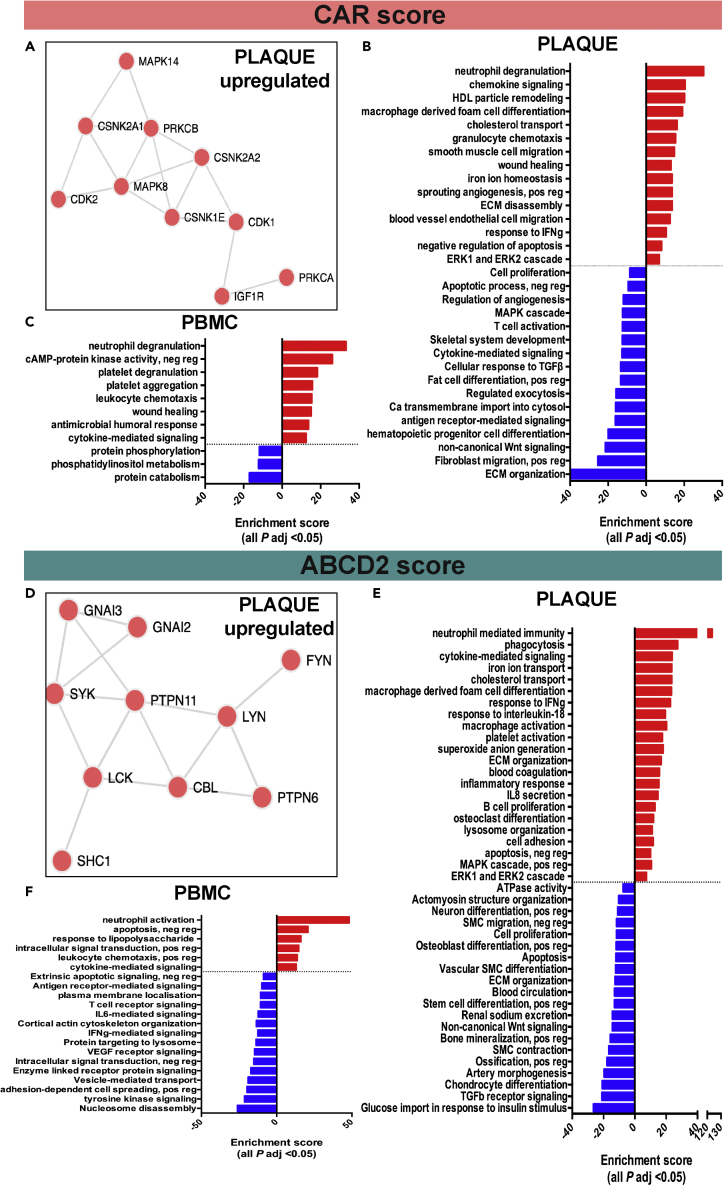
High CAR and ABCD2 scores associate with biological processes signifying plaque instability See also Tables S4 and S9. (A) Protein-protein interaction networks enriched among the significantly upregulated genes in plaques from high vs. low-risk CAR comparison. (B) Pathways induced in plaques from high-risk vs. low-risk patients in the CAR cohort were enriched for inflammation, foam cell differentiation, lipid transport, smooth muscle cell, endothelial cell migration, coagulation, and angiogenesis. Repressed pathways in the same comparison were enriched for extracellular matrix organization, fibroblast migration, exocytosis, and progenitor cell differentiation. (C) Similar analyses for PBMCs revealed induction of several pathways involving platelets and inflammation, as well as regulation of wound healing. Among the repressed pathways in PBMCs, protein phosphorylation and cell-cycle regulation were observed. (D) Protein-protein interaction networks enriched among significantly upregulated genes in plaques from high-risk vs. low-risk ABCD2 comparison. (E) In plaques from high-risk vs. low-risk patients in the ABCD2 cohort the top enriched pathway was neutrophil mediated immunity, in addition to pathways coupled to foam cell differentiation, cholesterol transport, and coagulation, whereas positive regulation of mineralization/ossification, chondrocyte differentiation, smooth muscle cell migration, and extracellular matrix organization were all repressed. (F) Similar analyses in PBMCs coupled high-risk to enriched inflammation, apoptosis, and intracellular signal transduction. Interestingly, among repressed pathways in PBMCs, IL-6 and VEGF signaling were found, in addition to regulation of cell proliferation and vesicle-mediated transport. Number of samples used in CAR analyses: plaques n = 58, PBMCs n = 44. Number of samples used in ABCD2 analyses: plaques n = 30, PBMCs n = 22.

Global gene expression analysis comparing plaques from patients with high vs. low-risk CAR score resulted in 1030 significantly differentially expressed genes, of which 576 genes were upregulated and 454 downregulated (Tables S2 and S3). Genes such as ion transporter *ABCB5*, scavenger receptor *CD36*, cytokines *GREM1* and *IL8*, oxidative stress-associated *SOD2,* and matrix metalloproteases (*MMP7, MMP8*) were among the most upregulated in high-risk CAR groups, whereas actin cytoskeleton and smooth muscle cells (SMCs)-associated *CDC42* and *PDLIM7* were among the downregulated genes. The key drivers of protein-protein interactions (PPI) predicted on the basis of significantly upregulated genes from this comparison were related to the regulation of cell cycle, differentiation, and apoptosis (*CSNK2A1, CSNK2A2, CSNK1E, CDK1, MAPK1, and MAPK14*) (Figure 3A and Table S4). Metabolites predicted to associate with this set of upregulated genes were related to iron, oxygen, and glycerol-phosphate metabolism (Table S4). Gene set enrichment and pathway analyses of significantly upregulated genes in plaques from the CAR group showed induction of classical atherosclerotic processes, such as chemokine- mediated signaling, inflammation, foam cell differentiation, lipid transport, SMC, and endothelial cell migration. Moreover, plaques from this group also showed processes coupled to instability, such as iron ion homeostasis (with genes such as *NFE2, ALAS2, TFRC, SLC11A2, HBD, CA2, CDC27, BMP2K, TSPAN5, TOP1, CTSB, and SNCA*), angiogenesis (*SPP1, STC, LPL, PDGFA, and PF4*), and coagulation (*ANXA1, MMP7, MMP1, MMP9, LGMN, CTSB*, *PF4, ITGB3, APOC1, and FN1*). Among the repressed pathways were ECM organization, exocytosis, and progenitor cell differentiation (Figure 3B). When comparing PBMCs from high vs. low CAR score patients, totally 879 genes were significantly differentially expressed, of which 356 were upregulated and 523 downregulated (Tables S5 and S6). Induced processes were again coupled to inflammatory responses and cytokine-mediated signaling, as well as platelet activation, degranulation, and aggregation, whereas cell cycle regulation and protein synthesis were repressed (Figure 3C).

### High ABCD2 score associates with neutrophil-mediated immunity, foam cell differentiation, cholesterol transport, and coagulation

Similar analyses were conducted in the ABCD2 group by comparing plaques from patients with high vs. low-risk ABCD2 scores. This resulted in 1885 significantly differentially expressed genes, of which 624 were upregulated and 1261 downregulated (Tables S7 and S8). Here, again *ABCB5*, bone matrix protein *IBSP*, heparan sulfate biosynthesis enzyme *HS3ST2*, leukocyte-activating proteins *CXCL5* and *CCL7*, and lipid metabolism-associated *PLD1* and *APOE* were strongly upregulated, whereas downregulated were SMC associated genes *MYOCD, MYH10, MYOZ2, ITGA8,* and *SOST* implicated in calcification. The key drivers of PPIs predicted from significantly upregulated genes in this comparison were mainly Src kinases involved in the regulation of innate and adaptive immune responses, hematopoiesis, and responses to growth factors and cytokines (*LYN, FYN, LCK,* and *SYK*) (Figure 3D and Table S9). Metabolites predicted to associate with this set of upregulated genes were, as in CAR analysis, related to iron and oxygen as common factors but also cholesterol and statin metabolism (Table S9). The top enriched pathways among the upregulated genes were neutrophil-mediated immunity, foam cell differentiation, cholesterol transport, and coagulation (*ANXA1, MMP8, LGMN, CTSB, PF4, APOC1, FN1, PECAM1,* and *PLAU*), whereas positive regulation of mineralization/ossification, chondrocyte differentiation, SMC migration, and ECM organization were overall repressed (Figure 3E). In PBMCs, a total of 3318 genes were significantly dysregulated in gene expression analysis comparing high score vs. low score, with 1986 upregulated and 1332 downregulated genes (Tables S10 and S11). High ABCD2 score was coupled to increased inflammation and apoptosis. Interestingly, among the repressed pathways were IL6 and VEGF signaling, along with regulation of cell proliferation and vesicle-mediated transport (Figure 3F).

### Biological processes signifying ossification and SMC proliferation are enriched in high CAR compared to high ABCD2 risk plaques

In addition, a direct comparison of plaques from high-risk CAR vs. high-risk ABCD2 patients showed that pathways like endothelial cell apoptosis, ossification, chondrocyte differentiation, and SMC proliferation were induced, whereas pathways associated with secretion and antigen presentation via MHC-II were repressed (Figure S1).

In the opposite comparison of low-risk CAR vs. low-risk ABCD2 plaques, induced pathways were negative regulation of leukocyte and macrophage differentiation, positive regulation of IL-2 production, and inflammatory response. Among the repressed pathways were SMC and stem cell differentiation, artery morphogenesis, and ossification (Figure S2).

### *ABCB5* is upregulated in plaques from patients with high CAR and ABCD2 risk and localized to CD68^+^ macrophages around neovessels and IPH

**Figure 4:**
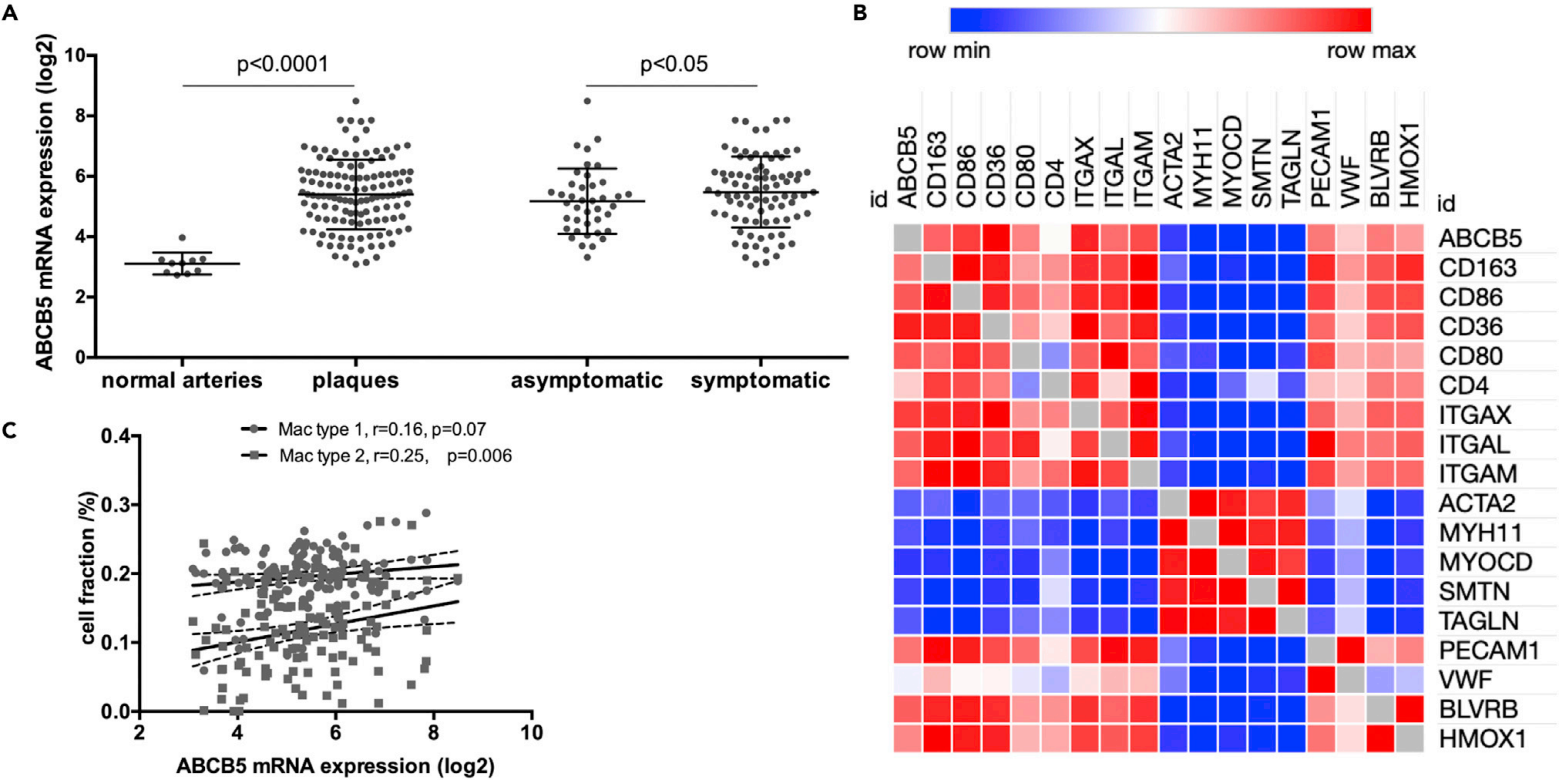
*ABCB5* is a previously unexplored atherosclerosis-related gene, upregulated in plaques from patients with high CAR and ABCD2 risk See also Tables S2, S7 and S12. *ABCB5* was one of the most upregulated genes in plaques from high-risk patients both by CAR and ABCD2 scoring. (A) In an extended BiKE cohort, microarray analysis comparing plaques (n = 127) vs. normal arteries (n = 10) as well as plaques from symptomatic (n = 87) vs. asymptomatic (n = 40) patients, confirmed the strong upregulation of *ABCB5* in the disease. Data are presented as mean ± SD. (B) Expression correlation analysis from plaque microarrays showed that *ABCB5* associated positively with the established macrophage, lymphocyte, and endothelial markers, as well as markers of intra-plaque hemorrhage, whereas it was negatively associated with common markers of typical smooth muscle cells. (C) By deconvolution analyses, plaque mRNA expression of *ABCB5* also correlated positively particularly with type 2 macrophages.

A comparison of all significantly differentially expressed transcripts in plaques from CAR vs. ABCD2 risk analysis showed that 358 genes were commonly dysregulated (Table S12). Interestingly, ATP-binding cassette subfamily B member 5 (*ABCB5)* was one of the most significantly upregulated genes in plaques from both CAR and ABCD2 high-risk patients (Tables S2 and S7). This finding was validated in an extended cohort of patients by microarrays comparing carotid plaques (n = 127) vs. normal arteries (n = 10) (mean log2 difference with SD = 2.29 ± 0.15, p < 0.0001) but also by comparing plaques from symptomatic (n = 87) vs. asymptomatic (n = 40) patients (mean log2 difference with SD = 0.30 ± 0.21, p < 0.05) (Figure 4A).

To begin characterizing the possible role of *ABCB5* in plaques, its mRNA expression was then examined in relation to major cell types present in plaques (Figure 4B), showing strong positive correlations with *CD36, CD86, CD163,* and *CD80* markers of macrophages (all r > 0.50, p < 0.0001) and common lymphocyte markers such as *ITGAM, ITGAE, CD28,* and *ITGAL* (all r > 0.50, p < 0.0001). *ABCB5* also showed significant positive correlations with endothelial markers *PECAM1* (r = 0.46, p < 0.0001) and *VWF* (r = 0.26, p = 0.0034). Interestingly, *ABCB5* was significantly positively correlated with markers of iron metabolism and IPH; *BLVRB* (r = 0.45, p < 0.0001), and *HMOX1* (r = 0.37, p < 0.0001) (Matic et al., 2018). A bioinformatic approach utilizing deconvolution to enumerate the relative cell fractions in plaques revealed that *ABCB5* transcript correlated positively particularly with type 2 macrophage fraction (Figure 4C). With respect to typical SMC markers, *ABCB5* expression was significantly inversely correlated with *ACTA2, MYH11, MYOCD,* and *SMTN* (all r < −0.30, p < 0.0001).

**Figure 5:**
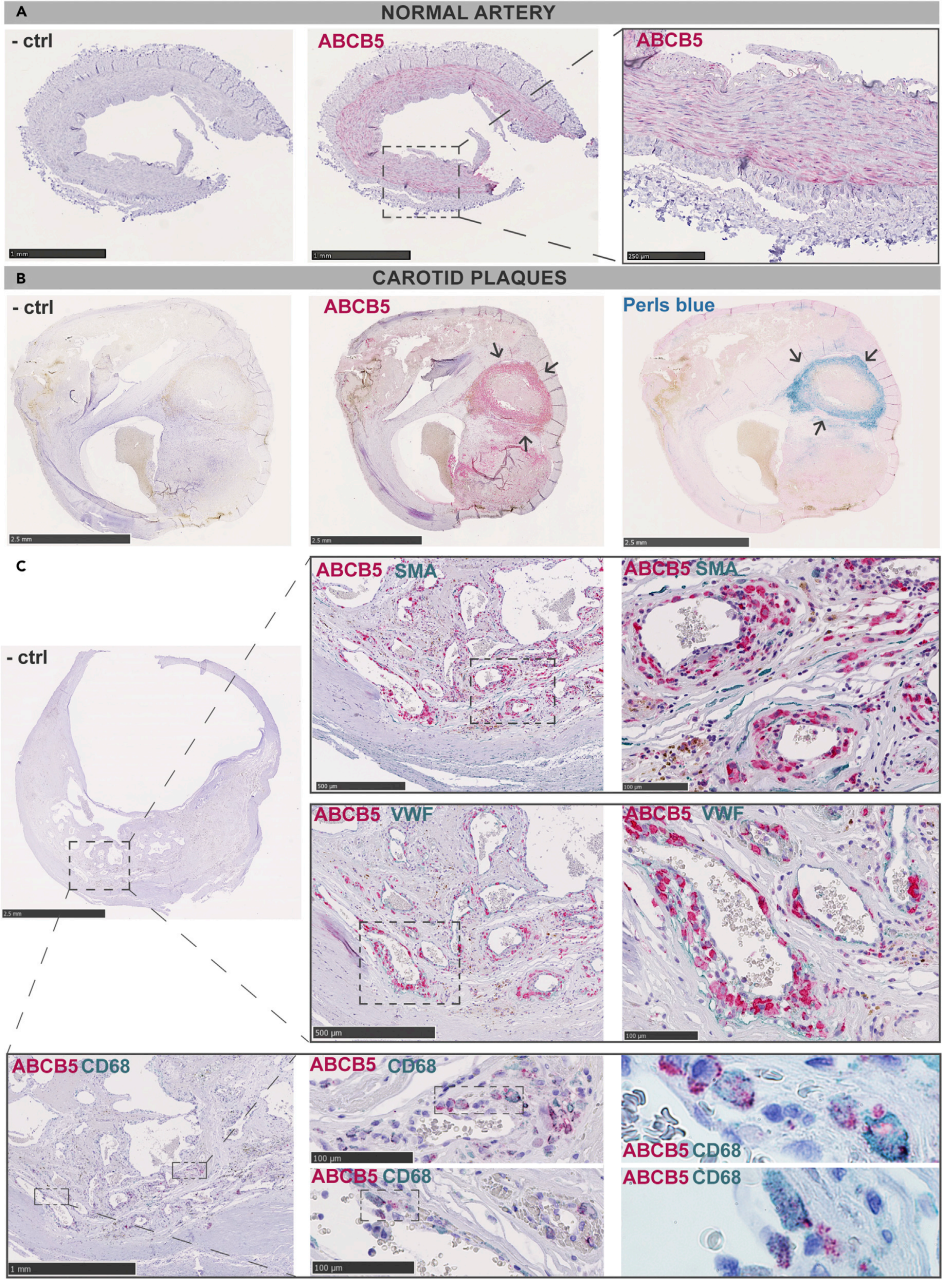
*ABCB5* is expressed in areas of intra-plaque hemorrhage in carotid plaques (A) Immunohistochemistry in normal arteries showed that *ABCB5* was expressed mainly in the medial layer. (B) In carotid plaques, abundant *ABCB5* expression was seen in the plaque necrotic core, especially in areas with intra-plaque hemorrhage as shown by Perl’s blue stain (arrows) (n = 3). (C) *ABCB5* expression was observed around the plaque neovessels, not overlapping with SMA + smooth muscle cells and VWF + endothelial cells in the same areas. Instead, *ABCB5* expression was shifted to CD68 ^+^ macrophages in these areas. Note the subcellular vesicular localization of *ABCB5* in these cells at higher magnifications. Images show representative stainings of plaques from n = 9 patients.

Immunohistochemistry was then used to evaluate the localization of *ABCB5* protein in human vascular tissues. In control normal arteries, *ABCB5* was detected in the medial layer (Figure 5A), whereas in carotid plaques the protein was found to be broadly abundant in the necrotic core, especially in areas with IPH as shown by Perl’s blue stain (Figure 5B). More detailed co-localization studies showed that strong *ABCB5* expression could be observed around the plaque neovessels but not overlapping with SMA + SMCs and VWF + endothelial cells in the same areas. Instead, *ABCB5* expression was localized to CD68 ^+^ macrophages in these areas, particularly in the subcellular vesicles (Figure 5C).

## Discussion

Here, we retrospectively performed clinical risk scoring for stroke of symptomatic CS patients who had undergone CEA, using ABCD2 and CAR tools. High-risk in patients estimated by these scores was attributed to molecular pathways in plaques and PBMCs typical for a vulnerable profile and unstable atherosclerosis, such as IPH, neovessel formation, inflammation, and foam cell differentiation. *ABCB5*, previously not described in the context of atherosclerosis, was one of the top genes identified by both high CAR and ABCD2 risk scores, localized to macrophages, IPH, and neovessels in plaques from symptomatic patients.

The primary goal of this study was to evaluate if symptomatic CS substratified by clinical risk scores can be associated with molecular transcriptomic signatures, which reflect pathophysiological processes in plaques and PBMCs. Both scoring systems correlated with the molecular pathways that were previously associated with vulnerability in symptomatic patients (Perisic et al., 2016) and revealed that ongoing inflammation, ECM reorganization, and coagulation were prominently upregulated pathways in plaques and PBMCs from high-risk patients. These findings were in agreement with the previous results from our group where transcriptomic and proteomic data were combined (Matic et al., 2018), showing that plaque profiles can discriminate between asymptomatic and symptomatic patients and even between symptomatic patients with TIA/minor stroke vs. amaurosis fugax (Perisic et al., 2016). Here, many similarities in comparison with those previous studies were seen, especially in the enriched pathways, such as inflammatory response, macrophage-derived foam cell differentiation, and ECM disassembly. In addition, there were similarities with a far smaller study where stroke-associated plaques where compared to asymptomatic plaques (Saksi et al., 2011). Multiple top upregulated genes were common, such as *CD36, HLA-DQA1, GLUL, IL8, MMP7, APOE,* and *SOD2* and *MYH10* among the downregulated genes. As expected, established pathways and genes related to atherosclerosis in general were also replicated in our study. However, we could also extend these results and demonstrate that plaques from high-risk symptomatic patients were more clearly enriched in pathways coupled to neovascularization, ongoing coagulation, angiogenesis, iron homeostasis, endothelial cell migration, and wound healing, all linked to neovessel formation and IPH, emphasizing a more severe state of plaque instability (Parma et al., 2017). Network analyses of the upregulated genes also highlighted the metabolism of iron as the predicted common factor for high-risk in both scoring systems.

Interestingly, the repressed pathways in symptomatic ABCD2 patients with high-risk scores were processes related to mineralization and ossification, and a direct comparison between high-risk CAR and high-risk ABCD2 patients again showed that ossification, chondrocyte differentiation, and SMC proliferation were relatively more prominent in association with the high CAR score. These pathways were previously reported by us as being upregulated in plaques from asymptomatic patients and patients on statin therapy (Perisic et al., 2016), which combined with results obtained in this study, raises the notion that calcification could be associated with a generally more stable molecular profile of the lesion, even in symptomatic patients (Karlof et al., 2019; Shaalan et al., 2004; Wahlgren et al., 2009).

*ABCB5*, a gene previously not studied in the context of atherosclerosis, emerged as one of the top upregulated in plaques from both high-risk CAR and ABCD2 patients. In concordance with the metabolic network analyses, *ABCB5* showed correlation to type 2 macrophages and enzymes *BLVRB* and *HMOX1* that are involved in Hb catabolism axis, IPH, and regulation of plaque instability processes particularly via type 2 macrophages (Matic et al., 2018). On protein level, *ABCB5* localized to macrophages in the necrotic core, especially in areas of IPH and in proximity to neovessels. So far, *ABCB5* has mostly been described in cancer, where it was associated with tumor invasiveness, metastasis, angiogenesis, and drug resistance (Cheung et al., 2011; Frank et al., 2005; Frank et al., 2011; Guo et al., 2018), supporting our findings for its role also in neovessel formation in the context of atherosclerotic plaque instability.

To provide additional support for further clinical risk stratification of symptomatic patients, ABCD2 and CAR scores were compared with each other as well as with clinical parameters representing plasma biomarkers. Despite the differences in parameters included in the respective scoring methods, these risk scores moderately correlated with each other, which rationalized our use of both methods for stratification of symptomatic patients. Analysis of laboratory blood values showed a correlation between high CAR score and higher levels of fibrinogen and WBC count, which may reflect active inflammation and coagulation because of plaque instability (Cao et al., 2013), especially because higher plasma fibrinogen levels have been associated with increased risk of coronary heart disease, stroke, and mortality (Danesh et al., 2005). In addition, these patients also had lower Hb levels, which has been suggested to be coupled to increased CVD risk (Kaiafa et al., 2015), and high-risk by ABCD2 also showed a trend toward lower Hb and higher fibrinogen levels. CAR score was also coupled to higher creatinine and lower eGFR, emphasizing the link between kidney disease and CVD (Pelisek et al., 2011; Wesseling et al., 2017). Both scoring systems showed negative correlation to HDL, which is expected considering that almost 90% of patients were on statins.

The risk scores have been developed in the early 2000s, but in the recent years, the capacity of ABCD2 to confidently stratify low-risk patients has been questioned (Wardlaw et al., 2015). Indeed, despite some modification attempts (Naylor et al., 2015), ABCD2 score is not recommended any more for use in the initial management of TIA to prevent referral delays for low-risk patients (National Institute for Health and Care Excellence, 2019 (NICE)). CAR was developed from ECST-I and is clinically only used in the frame of the ongoing ECST-II study. It is important to emphasize that our study was not aimed to evaluate the quality of these scoring systems; however, it is notable that even elementary stroke risk prediction tools can provide a further stratification in the group of symptomatic patients based on ongoing plaque vulnerability on a molecular level.

### Conclusion

To our knowledge, this is the first study assessing CAR and ABCD2 clinical risk scores for stroke in stratifying the underlying disease pathophysiology of patients with symptomatic carotid atherosclerosis. Our comprehensive analysis of plaques and PBMCs shows that both scoring methods can successfully capture the typical biological features of vulnerable patients and plaques such as inflammation, IPH, and neoangiogenesis. The stratification of symptomatic patients with high-risk was also corroborated by associations with laboratory parameters. The identified upregulation of *ABCB5* in plaques from patients scored as high-risk by both ABCD2 and CAR, emphasizes the significance of IPH in plaque instability, suggesting that the role of *ABCB5* in atherosclerosis should be mechanistically explored and its potential as a therapeutic target investigated. From a translational perspective, our study emphasizes the need for implementing more complex phenotyping for both plaques and patients by a combination of risk profiling and biomarkers that together may better reflect the underlying disease pathophysiology and improve stroke risk prediction.

### Limitations of the study

ABCD2 and CAR scores were developed two decades ago and neither of the risk scores are widely used clinically in the present time, with CAR score not yet validated and ABCD2 not recommended any more. ABCD2 lacks any input of the plaque imaging data, whereas CAR includes plaque stenosis degree and ulceration. However, the modern diagnostic selection process for surgery most often includes both data from carotid ultrasound, CTA, and in many cases also MRI. Although our study is small with respect to the correlations with clinical parameters, it should be regarded as extensive when it comes to comparing clinical risk scores to the underlying molecular profiles of corresponding patients. The BiKE cohort includes only patients with end-stage atherosclerosis and the results cannot be extrapolated to earlier stages of the disease. For molecular analyses, plaques were divided at surgery, where one half was used for histology and the other in transcriptomic profiling. This approach has obvious limitations, but the advantage is that the validation of the ongoing biology obtained from transcriptomic profiling can be confirmed with histological analyses in the matched plaque.

## STAR★Methods

### Key resources table

**Table:** 

REAGENT or RESOURCE	SOURCE	IDENTIFIER
**Antibodies**		

Anti-ABCB5 antibody produced in rabbit	Sigma-Aldrich	Cat# HPA026975; RRID: AB_10602007
Mouse CD68 antibody	Dako (now Agilent)	Cat# HPA026975; RRID: AB_10602007
Mouse von Willebrand Factor (vWF) antibody	Dako (now Agilent)	Cat# M0616; RRID: AB_2216702
Monoclonal Mouse Anti Human Smooth Muscle Actin (SMA) antibody	Dako (now Agilent)	Cat# M0851; RRID: AB_2223500

**Biological samples**		

Human Carotid Plaque Tissue	BiKE	N/A
Normal human arteries tissue	BiKE	N/A
Plasma from carotid patients	BiKE	NA

**Chemicals, peptides, and recombinant proteins**		

Perl’s stain kit	Atom Scientific	#RRSK16-100
DIVA buffer	BioCare Medical	DV2004
Warp Red	BioCare	WR806S
Vina Green	BioCare	MRR807AS
Background Sniper	BioCare	BS966L
MACH3 Rabbit AP-Polymer Detection kit	BioCare	M3R533H
MACH2 DoubleStain Cocktail Kit	BioCare	MRCT525
MACH3 mouse AP-Polymer Detection kit	BioCare	M3M532H

**Deposited data**		

Plaque microarrays (Affymetrix HGU-133 plus 2.0 Genechip arrays)	Gene Expression Omnibus	GSE125771
PBMC microarrays (Affymetrix HGU-133 plus 2.0 Genechip arrays)	Gene Expression Omnibus	GSE21545

**Software and algorithms**		

CAR score application	https://www.sealedenvelope.com/car/	N/A
ABCD2 risk calculator	MD Calc (https://www.mdcalc.com/abcd2-score-tia)	N/A
Prism	GraphPad	N/A
GOrilla	http://cbl-gorilla.cs.technion.ac.il	N/A
REVIGO	http://revigo.irb.hr	N/A
ENRICHR	https://maayanlab.cloud/Enrichr/	N/A

**Other**		

N/A	N/A	N/A
N/A	N/A	N/A

### Resource availability

#### Lead contact

Further information, resources and reagents will be fulfilled upon reasonable request and should be directed to the lead contact, Ljubica Matic (ljubica.matic@ki.se).

#### Materials availability

This study did not generate new unique reagents.

### Experimental model and subject details

Patients undergoing CEA at the Department of Vascular Surgery, Karolinska University Hospital, Stockholm, Sweden were consecutively included in the Biobank of Karolinska Endarterectomies and clinical data recorded on admission. Carotid plaques and peripheral blood samples were collected at the CEA surgery. Human studies from BiKE were approved by the Ethical Review Board and follow the guidelines of the Declaration of Helsinki. All human samples and data in BiKE were collected with informed consent from patients or organ donors’ guardians. Tissue and blood sampling were conducted as part of the ordinary medical and surgical procedures and did not put the patients at unnecessary risk.

### Method details

#### Human carotid stenosis cohort

This was a retrospective study utilizing a sub-cohort of BiKE patients enrolled between 2002 and 2011, where the inclusion criteria were based on symptomatic CS, existing duplex ultrasound and/or carotid CTA and availability of microarray analyses from plaques and PBMCs (Perisic et al., 2016). Symptoms of plaque instability were defined in this cohort as *amaurosis fugax* (retinal TIA), TIA, minor stroke and major stroke. Patients without qualifying symptoms within 180 days prior to surgery were considered asymptomatic.

From the clinical database adjoined to BIKE, patient’s medications (i.e. platelet inhibitors, lipid-lowering medication, antihypertensives) and laboratory values (i.e. WBC, Hb, CRP, LDL, cholesterol, serum creatinine) at the time of surgery, were obtained. Records of routine preoperative duplex ultrasound (blinded independent examiner) and/or carotid CTAs were used for evaluation of carotid plaques.

#### CAR and ABCD2 risk scoring

The clinical risk for stroke was evaluated for each patient by CAR and ABCD2 scores, by the same author K.W. Clinical parameters for risk scoring were collected from medical records and scores obtained with the Smartphone applications ‘CAR score’ for CAR and ‘MDCalc’ for ABCD2 (Claiborne Johnston, 2007; European Carotid Surgery Trial, 2015a). In accordance with the definitions in the CAR application, TIA was defined as symptoms lasting less than 24 hours independent from imaging findings; amaurosis fugax and retinal infarction were defined as ipsilateral monocular symptoms; minor stroke was defined as stroke with signs and symptoms lasting 24 hours to 7 days; major stroke was defined as non-disabling stroke with residual signs present or persisting longer than 7 days (European Carotid Surgery Trial, 2015b).

ABCD2 estimates the risk for stroke in 2 days, 7 days and 90 days after having a TIA. For the ABCD2 risk calculator age, initial blood pressure, diabetes, clinical features and duration of the TIA were parameters taken into account. ABCD2 score 0-3 points was considered low risk, 4-5 points intermediate (moderate) risk and 6-7 points high risk for stroke (Claiborne Johnston, 2007). In this study, all symptomatic patients were risk scored by ABCD2 even though they had symptoms persisting longer than 24hrs.

CAR score estimates the rate of 5-year ipsilateral IS in recently symptomatic patients. CAR score takes into account sex, age, degree of CS, presence of near total vessel occlusion or not, time in days from the most recent event to treatment, most severe ipsilateral symptom, previous myocardial infarction, peripheral vascular disease, diabetes, hypertension and whether the plaque is ulcerated or not. In CAR score, the degree of stenosis is measured by the North American Symptomatic Carotid Endarterectomy Trial (NASCET)-criteria (Barnett et al., 1991) and when necessary, the recorded degree of stenosis was converted from ECST to NASCET criteria (Jogestrand, 2011). In the majority of imaging reports, the degree of stenosis was reported as a range (i.e. 70-99%) which was converted to an average (85%); near occlusion was defined as severe stenosis with distal collapse of the artery. The CAR score suggests the use of non-invasive imaging for estimating the presence of ulceration, even if conventional carotid angiograms were used in the development of the scoring tool. In this study, ulceration was only entered if there was clear evidence of ulcerated plaque on non-invasive imaging in accordance with the scoring instructions (European Carotid Surgery Trial, 2015b). For CAR, low risk was considered when score was 5-10%, intermediate risk had score 11-13% and high risk group had score 14-37% (estimation of future risk for stroke given in percentages).

#### Collection of human material and RNA extraction

Carotid plaques and peripheral blood samples were collected at surgery, and plaques were directly divided into two halves transversally at the most stenotic part by the surgeon. The proximal half of the lesion was frozen at −80°C immediately after surgery for RNA preparation, the distal half fixed in 4% Zn-formaldehyde and processed for histology. For controls, normal arteries were obtained from nine macroscopically disease-free iliac arteries and one aorta from organ donors without history of CVD. The normal vessels were dissected and the intima and media used for RNA isolation for microarrays. Whole blood was processed according to standard procedures for peripheral blood mononuclear cell fraction (PBMC) separation and isolation of RNA. Blood samples were centrifuged by density gradient through Ficoll-Paque preparation tubes (Vacutainer CPT, Becton-Dickson, Franklin Lakes, NJ, USA). PBMCs were resuspended using RLT buffer (Qiagen, Hilden, Germany) before freezing at −80°C. RNA from plaques and PBMCs was prepared using Qiazol Lysis Reagent (Qiagen) and purified using the RNeasy Mini kit (74106, Qiagen), including DNase digestion. The concentration of RNA was measured using Nanodrop ND-1000 (Thermo Scientific, Waltham, MA, USA) and quality estimated using Bioanalyser capillary electrophoreses system (Agilent Technologies, Santa Clara, CA, USA). All human samples were collected with informed consent from patients, organ donors or their guardians.

#### Gene expression analysis by microarrays

RNA from normal arteries, plaques and PBMCs was analyzed by gene expression microarrays. According to recommended standards, only RNA of good integrity (RNA Integrity Number >7), A260/A280 ratio 1.8-2.1, A260/A230 ratio 0.7-1.5 and concentration about 50-500 ng/μL was used. Gene expression analyses were performed using Affymetrix HGU-133 plus 2.0 Genechip arrays (Santa Clara, CA, USA). Robust multiarray average normalization, filtering of probe sets based on signal intensity and batch effect correction were performed, and processed gene expression data were recorded on a log2 scale (Perisic et al., 2016). The full data set is available from Gene Expression Omnibus (accession number GSE125771 and GSE21545).

#### Study setting

Of the 101 symptomatic carotid stenosis patients who had undergone CEA and had available microarrays, 98 patients were eligible to risk score by CAR and 100 patients were eligible to risk score by ABCD2. Correlation analyses for CAR *vs.* ABCD2 and risk scores *vs.* laboratory values were performed where all available patients were included. For the differential gene expression analyses, the patients from both ABCD2 and CAR risk scores were stratified into different risk groups: low, intermediate and high risk groups. Here, the intermediate group was excluded and only well-separated low *vs.* high risk were compared. This strategy was applied for the purpose of strengthening the significance in the discovery bioinformatic analyses. Since ABCD2 is developed mainly for patients presenting with TIA, only plaques from patients with TIA or retinal TIA were considered in the gene expression analysis.

#### Histological and immunohistochemical analyses

The distal half of each plaque collected at surgery was fixed in 4% Zn-formaldehyde and processed for histology. Before staining, slides were deparaffinized and rehydrated with ethanol series. The presence of ferric iron deposits in plaques was assessed by Perl's Prussian blue stain on adjacent serial plaque sections, performed using Perl’s stain kit #RRSK16-100 (Atom Scientific, Hyde, United Kingdom) according to the manufacturers’ instructions.

For immunohistochemistry (IHC) all reagents were from Biocare Medical (Concord, CA). Isotype rabbit and mouse IgG were used as negative controls. Primary antibodies used in the study were ABCB5 (HPA026975, RRID: AB_10602007, Sigma-Aldrich) and antibodies for cell-specific markers were: smooth muscle α-actin (SMA, M0851, DAKO), CD68 (M0876, DAKO) and vWF (M0616, DAKO). Tissue sections 5μm in thickness were deparaffinized in HistolabClear and rehydrated in a graded series of ethanol concentrations. For antigen retrieval, slides were high-pressure boiled in DIVA buffer (pH 6.0). Background Sniper was used as blocking, next primary antibodies diluted in Da Vinci Green solution were applied and incubated at room temperature for 1h. A double-stain probe-polymer system was applied, with subsequent detection using Warp Red and Vina Green. Slides were counter stained with Haematoxylin QS (Vector Laboratories, Burlingame, CA, USA) before dehydrated and mounted with Pertex (Histolab, Gothenburg, Sweden). The overlap between these Warp Red and Vina Green dyes resulting from close co-localization of two proteins is observed as brown/grey signal. Slides were scanned using an automated SlideScanner system and images were captured with NDPview. A Nikon OPTIPHOT-2 microscope equipped with a digital camera was also used and images were and processed with NIS-Elements software.

### Quantification and statistical analysis

#### Bioinformatic and statistical data analyses

Patients were categorized to low, intermediate or high-risk according to their CAR and ABCD2 scores mentioned above. Transcriptomic dataset analyses and statistical analyses were performed with GraphPad Prism. For demographics tables, two-sided Chi-square test was performed for nominal data and two-sided unpaired t-test for comparing the equality of groups. Continuous variables were summarized by their means, and categorical variables as counts and percentages. Distribution of the data was assessed using the Shapiro-Wilks normality test. A linear regression model adjusted for age and gender and a two-sided parametric Student’s t-test with correction for multiple comparisons according to Bonferroni, was used for microarray dataset analyses. Pearson rank correlations were calculated to determine the association between risk scores and clinical parameters, as well as between mRNA expression levels from microarrays.

Following analyses of differential gene expression, gene set enrichment analyses were performed on Gene Ontology (GO) terms using public software for comparison: GOrilla (http://cbl-gorilla.cs.technion.ac.il) with filtering by REVIGO software (http://revigo.irb.hr), ENRICHR; KEGG 2019, Reactome 2016, GO Process 2018 and GO Function 2018 (http://amp.pharm.mssm.edu/Enrichr). Categories with more than 3 genes and non-overlapping gene sets were considered in these analyses. Final data presented was based on ENRICHR GO Process 2018. ENRICHR software was also used for analyses of enriched PPIs, predicted cell signatures (based on Human Cell Atlas) and predicted metabolites (HMDB database) based on the upregulated genes in high *vs*. low risk CAR and ABCD2 comparisons.

Deconvolution of the BiKE microarray data was performed using a previously described (Wirka et al., 2019) single-cell RNA sequencing dataset from n = 4 coronary plaques *via* the Cibersort software (https://cibersort.standford.edu) (Newman et al., 2015). In brief, cell populations were defined using pre-assigned “cell-type signature markers” (top 26 genes specific for each of the major cell types) (Wirka et al., 2019) and used to specifically estimate the relative ratios of macrophage cell populations in the BiKE microarray data in association with *ABCB5* gene expression. Human Protein Atlas (www.proteinatlas.org), Gene Cards www.genecards.org and PubMed search were performed for description of gene function. In all bioinformatic analyses, adjusted p-values <0.05 were considered statistically significant.

## Data Availability

• Data: The full microarray dataset has been deposited at NCBI Gene Expression Omnibus and is publicly available. Accession numbers are listed in the key resources table. The individual human data reported in this study cannot be deposited in a public repository because of the GDPR and ethics laws that regulate the privacy of individuals that participated in the study. Other data reported in this paper will be shared by the lead contact upon a reasonable request. • Code: This paper does not report original code. • Other items: Any additional information required to reanalyze the data reported in this paper is available from the lead contact upon request.
