# Supplementary material for: Clinical risk scores for stroke correlate with molecular signatures of vulnerability in symptomatic carotid patients
Source: iScience. 2022 Apr 8;25(5):104219. doi: 10.1016/j.isci.2022.104219 (PMC9046225; doi:10.1016/j.isci.2022.104219)
Supplement: Document S1. Figures S1, S2 and Tables S1–S12 [file mmc1.pdf]

iScience, Volume *25*

Supplemental information

Clinical risk scores for stroke correlate with molecular signatures of vulnerability in symptomatic carotid patients

Katarina Wadén, Eva Karlöf, Sampath Narayanan, Mariette Lengquist, Göran K. Hansson, Ulf Hedin, Joy Roy, and Ljubica Matic

Figure S1

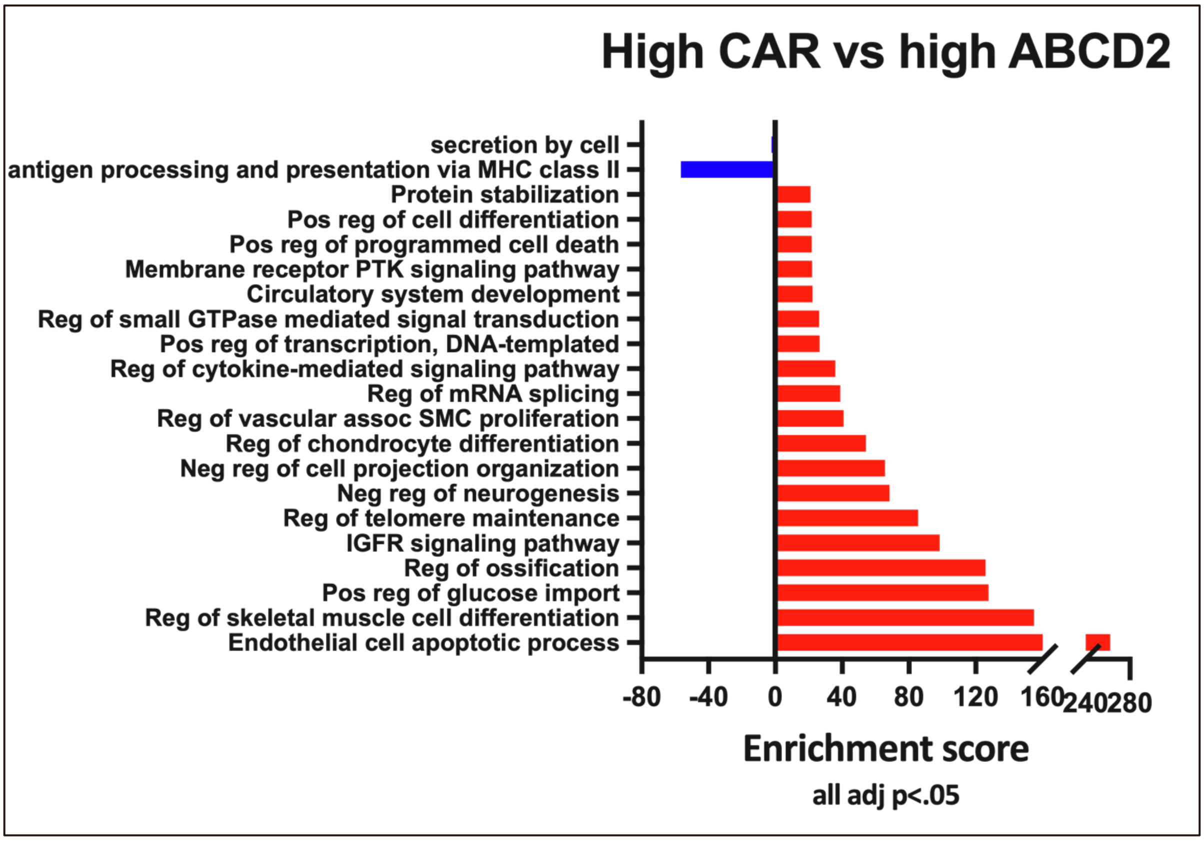

Figure S1. Gene set enrichment analysis of high CAR *vs.* high ABCD2 risk plaques, related to Figure 3. Enriched pathways in comparison of plaques from high risk CAR patients with high risk ABCD2 patients.

Figure S2

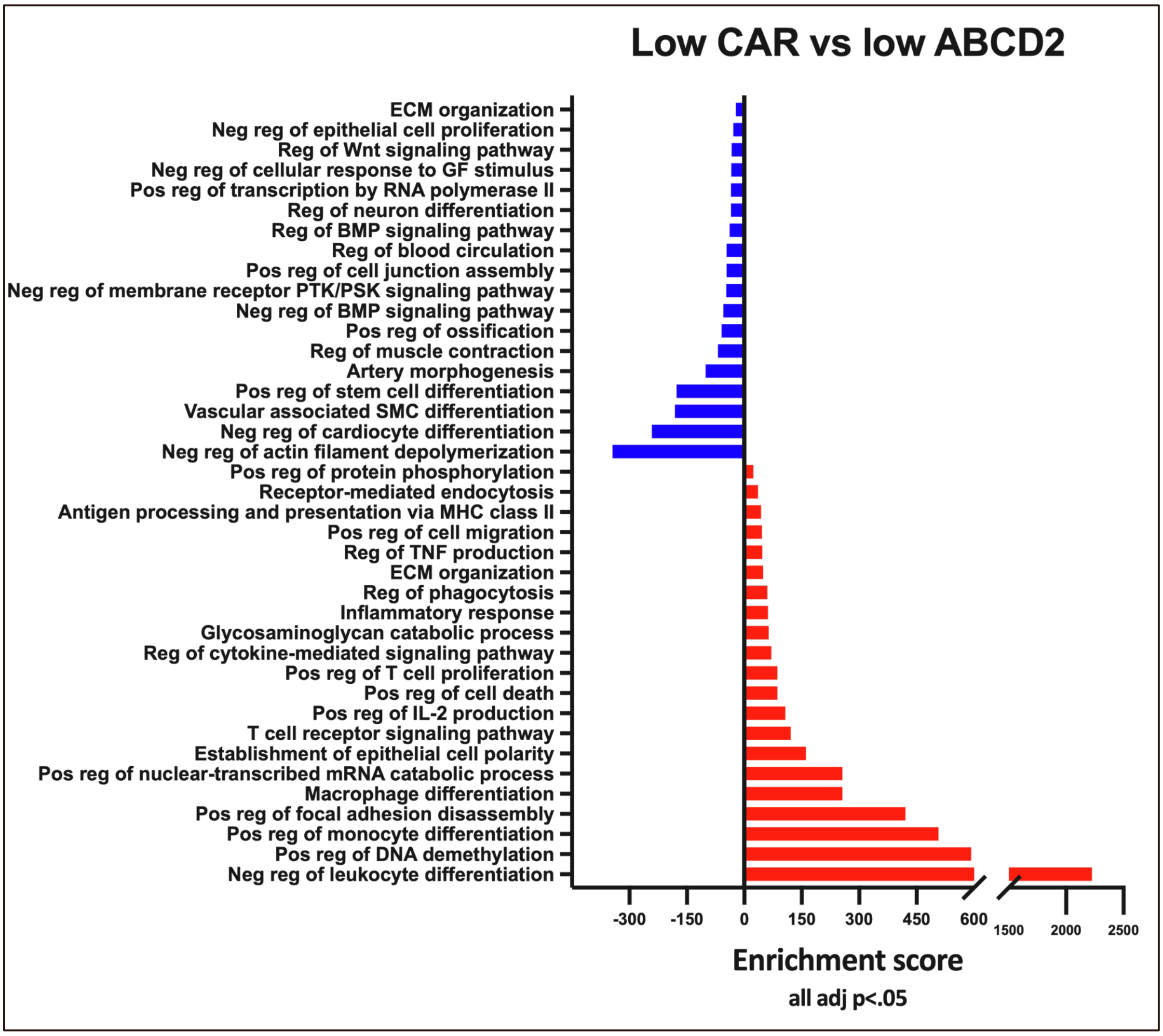

Figure S2. Gene set enrichment analysis of low CAR *vs.* low ABCD2 risk plaques, related to Figure 3. Enriched pathways in comparison of plaques from low risk CAR patients with low risk ABCD2 patients.

Table S1

	CAR[a]	ABCD2
Sex	CAR	
Age	CAR	+
Symptoms	CAR	+
Duration of symptoms		+
Time from event	CAR	
Hypertension	CAR	+
History of myocardial infarction	CAR	
History of Diabetes	CAR	+
Peripheral vascular disease	CAR	
Stenosis grade (NASCET[b])	CAR	
Near occlusion	CAR	
Ulceration	CAR	

Table S1. Scoring parameters used for CAR and ABCD2, related to STAR Methods and Figure 1. A list of all features used for clinical risk scoring in both groups as indicated by the Smartphone application.

[a] CAR = Carotid Artery Risk score

[b] NASCET = North American Symptomatic Carotid Endarterectomy Trial

Table S2

Gene Symbol	Gene Name	P-value	Fold change (upregulated)
ABCB5	ATP-binding cassette. sub-family B (MDR/TAP). member 5	< 0.0001	1.618
MALAT1	Metastasis associated lung adenocarcinoma transcript 1	< 0.0001	1.607
HLA-DQA1	Major histocompatibility complex. class II. DQ alpha 1	< 0.0001	1.563
ABCB5	ATP-binding cassette. sub-family B (MDR/TAP). member 5	< 0.0001	1.541
NR4A2	Nuclear receptor subfamily 4. group A. member 2	< 0.0001	1.505
SMC3	Structural maintenance of chromosomes 3	< 0.0001	1.498
JAK1	Janus kinase 1	< 0.0001	1.471
CD36	CD36 molecule (thrombospondin receptor)	< 0.0001	1.453
CD36	CD36 molecule (thrombospondin receptor)	< 0.0001	1.453
SMARCC1	SWI/SNF related matrix assoc actin dependent reg of chromatin, subfam c, member 1	< 0.0001	1.451
AP3D1	Adaptor-related protein complex 3. delta 1 subunit	< 0.0001	1.439
NR4A2	Nuclear receptor subfamily 4. group A. member 2	< 0.0001	1.434
SPRY4	Sprouty homolog 4 (Drosophila)	< 0.0001	1.426
SLC16A6	Solute carrier family 16. member 6 (monocarboxylic acid transporter 7)	< 0.0001	1.425
SLC16A10	Solute carrier family 16. member 10 (aromatic amino acid transporter)	< 0.0001	1.420
NR4A2	Nuclear receptor subfamily 4. group A. member 2	< 0.0001	1.417
U2AF1	U2 small nuclear RNA auxiliary factor 1	< 0.0001	1.416
MAP4	Microtubule-associated protein 4	< 0.0001	1.414
GREM1	Gremlin 1. cysteine knot superfamily. homolog (Xenopus laevis)	< 0.0001	1.413
VAT1L	Vesicle amine transport protein 1 homolog (T. californica)-like	< 0.0001	1.413
IL8	Interleukin 8	< 0.0001	1.411
CDC42BPA	CDC42 binding protein kinase alpha (DMPK-like)	< 0.0001	1.408
STK4	Serine/threonine kinase 4	< 0.0001	1.407
ANKRD11	Ankyrin repeat domain 11	< 0.0001	1.405
SOD2	Superoxide dismutase 2. mitochondrial	< 0.0001	1.400
PHACTR2	Phosphatase and actin regulator 2	< 0.0001	1.393
SLC2A3	Solute carrier family 2 (facilitated glucose transporter). member 3	< 0.0001	1.393
PF4	Platelet factor 4	< 0.0001	1.386
PROK2	Prokineticin 2	< 0.0001	1.384
ANKRD12	Ankyrin repeat domain 12	< 0.0001	1.382
GLUL	Glutamate-ammonia ligase (glutamine synthetase)	< 0.0001	1.377
ITCH	Itchy E3 ubiquitin protein ligase homolog (mouse)	< 0.0001	1.375
C19orf33	Chromosome 19 open reading frame 33	< 0.0001	1.373
MMP7	Matrix metallopeptidase 7 (matrilysin. uterine)	< 0.0001	1.370
TUBB1	Tubulin. beta 1	< 0.0001	1.370
MMP8	Matrix metallopeptidase 8 (neutrophil collagenase)	< 0.0001	1.368
CCDC88A	Coiled-coil domain containing 88A	< 0.0001	1.368
UBXN4	UBX domain protein 4	< 0.0001	1.365
MALAT1	Metastasis associated lung adenocarcinoma transcript 1	< 0.0001	1.365
UGCG	UDP-glucose ceramide glucosyltransferase	< 0.0001	1.365
AMFR	Autocrine motility factor receptor	< 0.0001	1.363
STC1	Stanniocalcin 1	< 0.0001	1.363
HECTD1	HECT domain containing 1	< 0.0001	1.353
CTNNB1	Catenin (cadherin-associated protein). beta 1	< 0.0001	1.352
MMP8	Matrix metallopeptidase 8 (neutrophil collagenase)	< 0.0001	1.352
RNF145	Ring finger protein 145	< 0.0001	1.351

ERAP2	Endoplasmic reticulum aminopeptidase 2	< 0.0001	1.347
APOC1	Apolipoprotein C-I	< 0.0001	1.347
BOD1L	Biorientation of chromosomes in cell division 1-like	< 0.0001	1.345
ADM	Adrenomedullin	0.0001	1.344

Table S2. Related to Figure 3. List of top significantly upregulated genes comparing plaques from high *vs*. low risk CAR patients.

Table S3

Gene Symbol	Gene Name	P-value	Fold change (downregulated)
HLA-DQA1	Major histocompatibility complex. class II. DQ alpha 1	< 0.0001	-2.155
HLA-DQA1	Major histocompatibility complex. class II. DQ alpha 1	< 0.0001	-2.036
HLA-DQB1	Major histocompatibility complex. class II. DQ beta 1	< 0.0001	-1.901
CXCL13	Chemokine (C-X-C motif) ligand 13	< 0.0001	-1.616
MALAT1	Metastasis associated lung adenocarcinoma transcript 1	< 0.0001	-1.596
IGKC	Immunoglobulin kappa constant	< 0.0001	-1.538
IGJ	Immunoglobulin J polypeptide. linker protein for immunoglobulin alpha and mu polypeptides	< 0.0001	-1.528
CDC42	Cell division cycle 42 (GTP binding protein)	< 0.0001	-1.528
NFIB	Nuclear factor I/B	< 0.0001	-1.521
NTRK2	Neurotrophic tyrosine kinase. receptor. type 2	< 0.0001	-1.505
EPHX1	Epoxide hydrolase 1. microsomal (xenobiotic)	< 0.0001	-1.455
CD74	CD74 molecule. major histocompatibility complex. class II invariant chain	< 0.0001	-1.448
IGKV4-1	Immunoglobulin kappa variable 4-1	< 0.0001	-1.445
PGGT1B	Protein geranylgeranyltransferase type I. beta subunit	< 0.0001	-1.444
MGC29506	Hypothetical protein MGC29506	< 0.0001	-1.440
FAM20B	Family with sequence similarity 2. member B	< 0.0001	-1.439
IGL@	Immunoglobulin lambda locus	< 0.0001	-1.433
IGL@	Immunoglobulin lambda locus	< 0.0001	-1.426
CXCL9	Chemokine (C-X-C motif) ligand 9	< 0.0001	-1.421
PTGDS	Prostaglandin D2 synthase 21kDa (brain)	< 0.0001	-1.419
NR1D2	Nuclear receptor subfamily 1. group D. member 2	< 0.0001	-1.405
PTGDS	Prostaglandin D2 synthase 21kDa (brain)	< 0.0001	-1.403
IGKV1OR15-118	Immunoglobulin kappa variable 1/OR15-118 pseudogene	< 0.0001	-1.401
RIT1	Ras-like without CAAX 1	< 0.0001	-1.393
YWHAE	Tyrosine 3-monooxygenase/tryptophan 5-monooxygenase activation protein. epsilon polypeptide	< 0.0001	-1.389
PDLIM7	PDZ and LIM domain 7 (enigma)	< 0.0001	-1.378
IGHM	Immunoglobulin heavy constant mu	< 0.0001	-1.369
RBM6	RNA binding motif protein 6	< 0.0001	-1.368
IL32	Interleukin 32	< 0.0001	-1.366
CCDC88A	Coiled-coil domain containing 88A	< 0.0001	-1.365
MGC29506	Hypothetical protein MGC29506	< 0.0001	-1.363
TNRC6B	Trinucleotide repeat containing 6B	< 0.0001	-1.361
CALR	Calreticulin	< 0.0001	-1.359
CPNE3	Copine III	< 0.0001	-1.357
BANF1	Barrier to autointegration factor 1	< 0.0001	-1.355
PCGF1	Polycomb group ring finger 1	< 0.0001	-1.353
DOCK5	Dedicator of cytokinesis 5	< 0.0001	-1.353
RIT1	Ras-like without CAAX 1	< 0.0001	-1.352
HLA-DQB1	Major histocompatibility complex. class II. DQ beta 1	< 0.0001	-1.349
IGHD	Immunoglobulin heavy constant delta	< 0.0001	-1.348
WTAP	Wilms tumor 1 associated protein	< 0.0001	-1.347
ADAMTSL4	ADAMTS-like 4	< 0.0001	-1.346
IGL@	Immunoglobulin lambda locus	< 0.0001	-1.345
EBP	Emopamil binding protein (sterol isomerase)	<0.001	-1.344
NPR3	Natriuretic peptide receptor C/guanylate cyclase C (atrionatriuretic peptide receptor C)	<0.001	-1.343
CD79A	CD79a molecule. immunoglobulin-associated alpha	<0.001	-1.340
IGL@	Immunoglobulin lambda locus	<0.001	-1.339
RAP1GDS1	RAP1. GTP-GDP dissociation stimulator 1	<0.001	-1.338

PTGDS	Prostaglandin D2 synthase 21kDa (brain)	<0.001	-1.336
ITCH	Itchy E3 ubiquitin protein ligase homolog (mouse)	<0.001	-1.331

Table S3. Related to Figure 3. List of top significantly downregulated genes comparing plaques from high *vs*. low risk CAR patients.

Table S4

CAR protein-protein interactions predicted from upregulated genes (p<0.05)		
**Gene Name**	**P-value**	**Combined score**
CSNK2A1	6.911e-15	139.02
CSNK2A2	7.545e-8	65.20
IGF1R	0.00004367	34.92
PRKCA	0.00001282	28.71
CSNK1E	0.0001889	28.59
CDK1	0.00001236	27.10
MAPK8	0.00007963	26.01
PRKCB	0.0001308	24.40
RNPS1	0.00110	22.02
MAOK14	0.00009707	21.58
**CAR metabolites predicted from upregulated genes (p<0.05)**		
**Name**	**P-value**	**Combined score**
Fructose-6-phosphate	0.0005310	98.69
Glycerol	0.001489	61.52
Iron	0.00004581	36.38
2-phosphoglyceric acid	0.02561	34.50
1.2-dihexadecanoyl-sn-glycero-3-phosposerine	0.03027	29.63
1-O-phophono-D-glucopyranose	0.03027	29.63
Sodium heparin ($C_{26}H_{41}NO_{34}S_4$)	0.02159	21.22
Pyridxal 5'-phosphate	0.01832	13.94
Oxygen	0.007648	13.48
Copper	0.02827	13.17

Table S4. Related to Figure 3. List of top significantly enriched protein-protein interactions and metabolites predicted from upregulated genes comparing high *vs*. low risk CAR patients.

Table S5

Gene Symbol	Gene Name	P-value	Fold change (up regulated)
MGC13005	Hypothetical LOC84771	1.97E-07	1.561
TMEM45A	Transmembrane protein 45A	3.11E-07	1.550
DEFA4	Defensin. alpha 4. corticostatin	8.54E-07	1.524
HLA-DRB4	Major histocompatibility complex. class II. DR B4	1.35E-06	1.512
MLH3	MutL homolog 3 (E. coli)	1.57E-06	1.509
PTPRC	Protein tyrosine phosphatase. receptor type. C	1.83E-06	1.505
CD9	CD9 molecule	3.04E-06	1.491
HIST1H2AE	Histone cluster 1. H2ae	6.66E-06	1.471
NUMA1	Nuclear mitotic apparatus protein 1	1.15E-05	1.456
SLC35D3	Solute carrier family 35. member D3	1.15E-05	1.456
COCH	Coagulation factor C homolog. cochlin	1.64E-05	1.446
TNNT1	Troponin T type 1 (skeletal. slow)	2.26E-05	1.437
SCGB1C1	Secretoglobin. family 1C. member 1	3.19E-05	1.428
LTF	Lactotransferrin	3.62E-05	1.424
HLA-DRB4	Major histocompatibility complex. class II. DR B4	4.90E-05	1.416
IL1B	Interleukin 1. beta	5.01E-05	1.415
IL1B	Interleukin 1. beta	9.00E-05	1.398
LCN2	Lipocalin 2	9.61E-05	1.397
CLECL1	C-type lectin-like 1	9.79E-05	1.396
FOLR3	Folate receptor 3 (gamma)	1.24E-04	1.389
MLH3	MutL homolog 3 (E. coli)	1.26E-04	1.389
DSP	Desmoplakin	1.81E-04	1.378
MLH3	MutL homolog 3 (E. coli)	2.29E-04	1.371
HIST1H3H	Histone cluster 1. H3h	2.93E-04	1.364
MFAP3L	Microfibrillar-associated protein 3-like	3.45E-04	1.359
ST20	Suppressor of tumorigenicity 20	3.52E-04	1.358
PSD3	Pleckstrin and Sec7 domain containing 3	3.57E-04	1.358
P2RY12	Purinergic receptor P2Y. G-protein coupled. 12	3.76E-04	1.356
GGTA1	Glycoprotein. alpha-galactosyltransferase 1	4.10E-04	1.353
CTTN	Cortactin	4.27E-04	1.352
RHOBTB1	Rho-related BTB domain containing 1	4.71E-04	1.349
C9orf130	Chromosome 9 open reading frame 130	5.55E-04	1.344
CTTN	Cortactin	5.89E-04	1.342
TUBB2A	Tubulin. beta 2A	6.03E-04	1.341
HBD	Hemoglobin. delta	6.32E-04	1.340
RNF144B	Ring finger protein 144B	6.36E-04	1.340
C2orf88	Chromosome 2 open reading frame 88	7.845E-04	1.333
C19orf33	Chromosome 19 open reading frame 33	8.27E-04	1.332
BMP6	Bone morphogenetic protein 6	8.35E-04	1.331
STON2	Stonin 2	8.96E-04	1.329
RNF144B	Ring finger protein 144B	9.65E-04	1.327
GPR34	G protein-coupled receptor 34	9.91E-04	1.326
ADAM28	ADAM metallopeptidase domain 28	1.02E-03	1.325
CD200	CD200 molecule	1.04E-03	1.324
HIST1H2BJ	Histone cluster 1. H2bj	1.14E-03	1.321
SH3BGRL2	SH3 domain binding glutamic acid-rich protein like 2	1.19E-03	1.320
ERAP2	Endoplasmic reticulum aminopeptidase 2	1.21E-03	1.319
ELOVL7	ELOVL family member 7 (yeast)	1.22E-03	1.319
PTPRO	Protein tyrosine phosphatase. receptor type. O	1.25E-03	1.318
ALOX12	Arachidonate 12-lipoxygenase	1.27E-03	1.318

Table S5. Related to Figure 3. List of top significantly upregulated genes comparing PBMCs from high *vs*. low risk CAR patients.

Table S6

Gene Symbol	Gene Name	P value	Fold change (downregulated)
HLA-DQA1	Major histocompatibility complex. class II. DQ alpha 1	7.03E-35	-2.872
HLA-DQB1	Major histocompatibility complex. class II. DQ beta 1	1.31E-30	-2.677
HLA-DQA1	Major histocompatibility complex. class II. DQ alpha 1	1.27E-20	-2.220
HLA-DQB1	Major histocompatibility complex. class II. DQ beta 1	2.75E-11	-1.769
ZNF264	Zinc finger protein 264	5.04E-09	-1.650
RASA2	RAS p21 protein activator 2	1.78E-07	-1.564
ZCCHC2	Zinc finger. CCHC domain containing 2	2.19E-07	-1.559
RASA2	RAS p21 protein activator 2	9.47E-07	-1.522
FBXL20	F-box and leucine-rich repeat protein 20	2.13E-06	-1.501
ACBD3	Acyl-Coenzyme A binding domain containing 3	8.42E-06	-1.464
RPS26	Ribosomal protein S26	1.89E-05	-1.442
UHMK1	U2AF homology motif (UHM) kinase 1	3.19E-05	-1.428
FAM169A	Family with sequence similarity 169. member A	3.82E-05	-1.423
ZNF652	Zinc finger protein 652	3.85E-05	-1.423
UHMK1	U2AF homology motif (UHM) kinase 1	5.54E-05	-1.412
SLFN5	Schlafen family member 5	5.91E-05	-1.411
DYRK2	Dual-specificity tyrosine-(Y)-phosphorylation regulated kinase 2	7.15E-05	-1.405
XIST	X (inactive)-specific transcript (non-protein coding)	1.28E-04	-1.388
RAP2A	RAP2A. member of RAS oncogene family	1.40E-04	-1.386
GNLY	Granulysin	1.51E-04	-1.383
SPTBN5	Spectrin. beta. non-erythrocytic 5	1.52E-04	-1.383
SKI	V-ski sarcoma viral oncogene homolog (avian)	1.612E-04	-1.381
PRSS23	Protease. serine. 23	1.63E-04	-1.381
RAPGEF2	Rap guanine nucleotide exchange factor (GEF) 2	1.63E-04	-1.381
SAMD9L	Sterile alpha motif domain containing 9-like	1.99E-04	-1.375
RABGAP1L	RAB GTPase activating protein 1-like	2.16E-04	-1.373
LNPEP	Leucyl/cystinyl aminopeptidase	2.21E-04	-1.372
SCD5	Stearoyl-CoA desaturase 5	2.55E-04	-1.368
PLEKHA2	Pleckstrin homology domain containing. family A (phosphoinositide binding specific) member 2	2.68E-04	-1.366
GSTM4	Glutathione S-transferase mu 4	2.68E-04	-1.366
NUAK1	NUAK family. SNF1-like kinase. 1	3.05E-04	-1.362
GNLY	Granulysin	3.23E-04	-1.361
PRSS23	Protease. serine. 23	3.26E-04	-1.360
PCYOX1	Prenylcysteine oxidase 1	3.35E-04	-1.360
ZBTB38	Zinc finger and BTB domain containing 38	3.38E-04	-1.359
NRIP1	Nuclear receptor interacting protein 1	3.63E-04	-1.357
RORA	RAR-related orphan receptor A	3.95E-04	-1.355
IQGAP1	IQ motif containing GTPase activating protein 1	4.73E-04	-1.349
DNAJC3	DnaJ (Hsp40) homolog. subfamily C. member 3	4.75E-04	-1.349
ATM	Ataxia telangiectasia mutated	4.75E-04	-1.349
BAG5	BCL2-associated athanogene 5	5.26E-04	-1.346
HELB	Helicase (DNA) B	5.82E-04	-1.343
SNAP29	Synaptosomal-associated protein. 29kDa	5.99E-04	-1.342
SLFN5	Schlafen Family Member 5	0.0006	-1.342
ITCH	Itchy E3 Ubiquitin Protein Ligase	0.0006	-1.341
YME1L1	YME1 Like 1 ATPase	0.0007	-1.339
BTBD7	BTB Domain Containing 7	0.0007	-1.337
MED1	Mediator Complex subunit 1	0.0007	-1.337
PPM1K	Protein Phosphatase, Mg2+/Mn2+ Dependent 1K	0.0007	-1.337
PPPDE1	Desumoylating Isopeptidase 2	0.0008	-1.334

Table S6. Related to Figure 3. List of top significantly downregulated genes comparing PBMCs from high *vs*. low risk CAR patients.

Table S7

Gene Symbol	Gene Name	P-value	Fold change (upregulated)
IBSP	Integrin-binding sialoprotein	< 0.0001	1.900
ABCB5	ATP-binding cassette. sub-family B (MDR/TAP). member 5	< 0.0001	1.804
HS3ST2	Heparan sulfate (glucosamine) 3-O-sulfotransferase 2	< 0.0001	1.759
HLA-DQA1	Major histocompatibility complex. class II. DQ alpha 1	< 0.0001	1.716
SULT1C2	Sulfotransferase family. cytosolic. 1C. member 2	< 0.0001	1.714
ABCB5	ATP-binding cassette. sub-family B (MDR/TAP). member 5	< 0.0001	1.702
CLEC4G	C-type lectin domain family 4. member G	< 0.0001	1.652
NAPSB	Napsin B aspartic peptidase pseudogene	< 0.0001	1.624
NPL	N-acetylneuraminate pyruvate lyase (dihydrodipicolinate synthase)	< 0.0001	1.588
CXCL5	Chemokine (C-X-C motif) ligand 5	< 0.0001	1.588
HSPA6	Heat shock 70kDa protein 6 (HSP70B')	< 0.0001	1.584
BEAN	Brain expressed. associated with Nedd4	< 0.0001	1.583
NAPSB	Napsin B aspartic peptidase pseudogene	< 0.0001	1.573
SNCA	Synuclein. alpha (non A4 component of amyloid precursor)	< 0.0001	1.550
PKIB	Protein kinase (cAMP-dependent. catalytic) inhibitor beta	< 0.0001	1.549
GLB1L	Galactosidase. beta 1-like	< 0.0001	1.543
IRX1	Iroquois homeobox 1	< 0.0001	1.543
SULT1C2	Sulfotransferase family. cytosolic. 1C. member 2	< 0.0001	1.523
RARRES1	Retinoic acid receptor responder (tazarotene induced) 1	< 0.0001	1.527
CCL7	Chemokine (C-C motif) ligand 7	< 0.0001	1.525
CUGBP2	CUG triplet repeat. RNA binding protein 2	< 0.0001	1.525
NUMA1	Nuclear mitotic apparatus protein 1	< 0.0001	1.523
COL11A1	Collagen. type XI. alpha 1	< 0.0001	1.519
CFD	Complement factor D (adipsin)	< 0.0001	1.516
CD300LF	CD300 molecule-like family member f	< 0.0001	1.515
UBASH3B	Ubiquitin associated and SH3 domain containing. B	< 0.0001	1.514
IL8RB	Interleukin 8 receptor. beta	< 0.0001	1.514
IBSP	Integrin-binding sialoprotein	< 0.0001	1.513
PRPF18	PRP18 pre-mRNA processing factor 18 homolog (S. cerevisiae)	< 0.0001	1.512
PSPH	Phosphoserine phosphatase	< 0.0001	1.504
PF4V1	Platelet factor 4 variant 1	<0.001	1.499
PION	Pigeon homolog (Drosophila)	<0.001	1.496
PTX3	Pentraxin-related gene. rapidly induced by IL-1 beta	<0.001	1.491
GABRB2	Gamma-aminobutyric acid (GABA) A receptor. beta 2	<0.001	1.489
SIRPB1	Signal-regulatory protein beta 1	<0.001	1.475
PLD1	Phospholipase D1. phosphatidylcholine-specific	<0.001	1.460
CPM	Carboxypeptidase M	<0.001	1.453
AQP9	Aquaporin 9	<0.001	1.451
ALK	Anaplastic lymphoma receptor tyrosine kinase	<0.001	1.451
ATP6V0D2	ATPase. H+ transporting. lysosomal 38kDa. V0 subunit d2	<0.001	1.450
HSPA6	Heat shock 70kDa protein 6 (HSP70B')	<0.001	1.442
LILRA2	Leukocyte immunoglobulin-like receptor. subfamily A (with TM domain). member 2	<0.001	1.440

APOE	Apolipoprotein E	<0.001	1.436
PDPN	Podoplanin	<0.001	1.435
PROK2	Prokineticin 2	<0.001	1.435
BCL2A1	BCL2-related protein A1	<0.001	1.434
ATP6V0D2	ATPase. H+ transporting. lysosomal 38kDa. V0 subunit d2	<0.001	1.433
PRR5L	Proline rich 5 like	<0.001	1.432
LILRA2	Leukocyte immunoglobulin-like receptor. subfamily A (with TM domain). member 2	<0.001	1.428
OSCAR	Osteoclast associated. immunoglobulin-like receptor	<0.001	1.427

Table S7. Related to Figure 3. List of top significantly upregulated genes comparing plaques from high *vs*. low risk ABCD2 patients.

Table S8

Gene Symbol	Gene Name	P value	Fold change (downregulated)
MYOCD	Myocardin	< 0.0001	-3.061
TPH1	Tryptophan hydroxylase 1	< 0.0001	-2.364
MAB21L2	Mab-21-like 2 (C. elegans)	< 0.0001	-2.270
NPR3	Natriuretic peptide receptor C/guanylate cyclase C	< 0.0001	-2.221
MYH10	Myosin. heavy chain 1. non-muscle	< 0.0001	-2.218
MYOZ2	Myozenin 2	< 0.0001	-1.996
TMEM47	Transmembrane protein 47	< 0.0001	-1.994
CNTN4	Contactin 4	< 0.0001	-1.931
MYOZ2	Myozenin 2	< 0.0001	-1.908
PLD5	Phospholipase D family. member 5	< 0.0001	-1.893
NBLA00301	Nbla00301	< 0.0001	-1.846
MALAT1	Metastasis associated lung adenocarcinoma transcript	< 0.0001	-1.836
CP	Ceruloplasmin (ferroxidase)	< 0.0001	-1.825
ASPN	Asporin	< 0.0001	-1.820
SLC16A7	Solute carrier family 16. member 7	< 0.0001	-1.819
BMPR1B	Bone morphogenetic protein receptor. type IB	< 0.0001	-1.810
NR1D2	Nuclear receptor subfamily 1. group D. member 2	< 0.0001	-1.806
PDZRN3	PDZ domain containing ring finger 3	< 0.0001	-1.787
FAM19A5	Family with sequence similarity 19 member A5	< 0.0001	-1.771
NBLA00301	Nbla00301	< 0.0001	-1.752
NEXN	Nexilin (F actin binding protein)	< 0.0001	-1.747
PTPRD	Protein tyrosine phosphatase. receptor type. D	< 0.0001	-1.746
CALD1	Caldesmon 1	< 0.0001	-1.745
C8orf84	Chromosome 8 open reading frame 84	< 0.0001	-1.735
C8orf84	Chromosome 8 open reading frame 84	< 0.0001	-1.734
C8orf84	Chromosome 8 open reading frame 84	< 0.0001	-1.721
DLX2	Distal-less homeobox 2	< 0.0001	-1.720
RNF160	Ring finger protein 160	< 0.0001	-1.714
ITGA8	Integrin. alpha 8	< 0.0001	-1.711
SFRP1	Secreted frizzled-related protein 1	< 0.0001	-1.708
HAPLN1	Hyaluronan and proteoglycan link protein 1	< 0.0001	-1.708
FBXO32	F-box protein 32	< 0.0001	-1.708
SOST	Sclerosteosis	< 0.0001	-1.703
NEGR1	Neuronal growth regulator 1	< 0.0001	-1.700
FILIP1L	Filamin A interacting protein 1-like	< 0.0001	-1.699
UHMK1	U2AF homology motif (UHM) kinase 1	< 0.0001	-1.698
GOLIM4	Golgi integral membrane protein 4	< 0.0001	-1.690
ZEB1	Zinc finger E-box binding homeobox 1	< 0.0001	-1.687
IGL@	Immunoglobulin lambda locus	< 0.0001	-1.683
CNTN3	Contactin 3 (plasmacytoma associated)	< 0.0001	-1.678
SCARA3	Scavenger receptor class A. member 3	< 0.0001	-1.676
FHL5	Four and a half LIM domains 5	< 0.0001	-1.674
FIBIN	Fin bud initiation factor homolog (zebrafish)	< 0.0001	-1.669
CCRL1	Chemokine (C-C motif) receptor-like 1	< 0.0001	-1.663
OSR1	Odd-skipped related 1 (Drosophila)	< 0.0001	-1.660
FIBIN	Fin bud initiation factor homolog (zebrafish)	< 0.0001	-1.654
SCRG1	Stimulator of chondrogenesis 1	< 0.0001	-1.650
ANGPT1	Angiopoietin 1	< 0.0001	-1.647
MALAT1	Metastasis associated lung adenocarcinoma transcript 1	< 0.0001	-1.646
DNAJC3	DnaJ (Hsp40) homolog. subfamily C. member 3	< 0.0001	-1.645

Table S8. Related to Figure 3. List of top significantly downregulated genes comparing plaques from high *vs.* low risk ABCD2 patients.

Table S9

ABCD2 protein-protein interactions predicted from upregulated genes (p<0.05)		
**Gene Name**	**P-value**	**Combined score**
SYK	3.111e-8	103.02
LYN	1.345e-8	90.73
PTPN6	0.000004730	51.92
GNAI1	0.0002079	34.00
PTPN11	0.0002249	29.09
GNAI3	0.0009028	25.06
SHC1	0.002818	15.84
CBL	0.004378	14.52
LCK	0.008165	11.73
FYN	0.009217	8.87
**ABCD2 metabolites predicted from upregulated genes (p<0.05)**		
**Name**	**P-value**	**Combined score**
Cholesterol	0.004154	60.91
Fructose-6-phosphate	0.007355	42.86
Iron	0.00001892	40.80
Adenosine	0.01011	35.07
Pyruvic acid	0.005310	25.46
Simvastatin	0.02158	21.29
Arachidonic acid	0.008159	20.88
Sodium heparin ($C_{26}H_{41}NO_{34}S_4$)	0.02397	19.81
Oxygen	0.009799	12.27
Protoporphyrin IX containing Fe ($C_{34}H_{34}N_4O_4.Fe$)	0.02159	8.83

Table S9. Related to Figure 3. List of top significantly enriched protein-protein interactions and metabolites predicted from upregulated genes comparing high *vs*. low risk ABCD2 patients.

Table S10

Gene Symbol	Gene Name	P-value	Fold change (upregulated)
CLC	Charcot-Leyden crystal protein	2.51E-22	2.539
FOLR3	Folate receptor 3 (gamma)	1.46E-20	2.439
S100B	S100 calcium binding protein B	2.51E-18	2.310
MS4A3	Membrane-spanning 4-domains. subfamily A. member 3 (hematopoietic cell-specific)	1.44E-16	2.208
RNASE4	Ribonuclease. RNase A family. 4	6.48E-14	2.052
PTGS2	Prostaglandin-endoperoxide synthase 2 (prostaglandin G/H synthase and cyclooxygenase)	4.35E-13	2.003
MSR1	Macrophage scavenger receptor 1	6.36E-13	1.993
AREG	Amphiregulin	1.41E-12	1.972
FAM118A	Family with sequence similarity 118. member A	2.94E-12	1.953
DEFA4	Defensin. alpha 4. corticostatin	3.47E-12	1.949
USF1	Upstream transcription factor 1	1.33E-11	1.913
IL1B	Interleukin 1. beta	1.43E-11	1.911
SERPINB2	Serpin peptidase inhibitor. clade B (ovalbumin). member 2	2.07E-11	1.901
SYNM	Synemin. intermediate filament protein	3.38E-11	1.888
GPR34	G protein-coupled receptor 34	2.38E-10	1.836
FPR2	Formyl peptide receptor 2	6.08E-10	1.810
RGS1	Regulator of G-protein signaling 1	6.57E-10	1.808
SDC2	Syndecan 2	1.4E-09	1.787
HBB	Hemoglobin. beta	1.69E-09	1.782
DUSP1	Dual specificity phosphatase 1	1.74E-09	1.781
HBB	Hemoglobin. beta	1.75E-09	1.781
MCC	Mutated in colorectal cancers	2.26E-09	1.774
LTF	Lactotransferrin	2.34E-09	1.773
IL8	Interleukin 8	3.98E-09	1.758
NUMA1	Nuclear mitotic apparatus protein 1	4.35E-09	1.756
PSPH	Phosphoserine phosphatase	4.45E-09	1.755
KCNJ2	Potassium inwardly-rectifying channel. subfamily J. member 2	5.2E-09	1.751
CEACAM8	Carcinoembryonic antigen-related cell adhesion molecule 8	7.64E-09	1.740
FPR2	Formyl peptide receptor 2	7.93E-09	1.739
HBD	Hemoglobin. delta	1.07E-08	1.731
FH	Fumarate hydratase	1.3E-08	1.725
SGMS2	Sphingomyelin synthase 2	1.31E-08	1.725
KYNU	Kynureninase (L-kynurenine hydrolase)	1.67E-08	1.718
ANG	Angiogenin. ribonuclease. RNase A family. 5	1.94E-08	1.714
MS4A3	Membrane-spanning 4-domains. subfamily A. member 3	2.28E-08	1.709
LILRA5	Leukocyte immunoglobulin-like receptor. subfamily A member 5	2.93E-08	1.702
EIF5A	Eukaryotic translation initiation factor 5A	3.41E-08	1.698
HBB	Hemoglobin. beta	3.79E-08	1.695
CPA3	Carboxypeptidase A3 (mast cell)	6.13E-08	1.681
AKAP12	A kinase (PRKA) anchor protein 12	7.19E-08	1.676
AMFR	Autocrine motility factor receptor	9.57E-08	1.668
IL1B	Interleukin 1. beta	1.31E-07	1.659
IL8	Interleukin 8	1.86E-07	1.648
THBS1	Thrombospondin 1	2.62E-07	1.638
PLA2G7	Phospholipase A2. group VII (platelet-activating factor acetylhydrolase. plasma)	2.77E-07	1.637
RTKN2	Rhotekin 2	2.81E-07	1.636

TMCC3	Transmembrane and coiled-coil domain family 3	3.83E-07	1.627
RGS1	Regulator of G-protein signaling 1	4.38E-07	1.623
THBS1	Thrombospondin 1	4.78E-07	1.621
CCR3	Chemokine (C-C motif) receptor 3	5.15E-07	1.618

Table S10. Related to Figure 3. List of top significantly upregulated genes comparing PBMCs from high *vs*. low risk ABCD2 patients.

Table S11

Gene Symbol	Gene Name	P-value	Fold change (downregulated)
MLL3	Myeloid/lymphoid or mixed-lineage leukemia 3	3.29E-19	-2.361
RPS27	Ribosomal protein S27	8.22E-17	-2.222
RNF213	Ring finger protein 213	1.03E-13	-2.040
CUGBP2	CUG triplet repeat. RNA binding protein 2	1.4E-13	-2.033
MALAT1	Metastasis associated lung adenocarcinoma transcript	1.85E-13	-2.025
SMCHD1	Structural maintenance of chromosomes flexible hinge domain containing 1	2.91E-13	-2.013
LRRFIP1	Leucine rich repeat (in FLII) interacting protein 1	1.92E-12	-1.964
UHMK1	U2AF homology motif (UHM) kinase 1	2.76E-12	-1.955
MLL	Myeloid/lymphoid or mixed-lineage leukemia	2.9E-12	-1.953
NEAT1	Nuclear paraspeckle assembly transcript 1 (non-protein coding)	1.13E-11	-1.917
GGNBP2	Gametogenetin binding protein 2	1.19E-11	-1.916
C21orf81	Ankyrin repeat domain 20 family. member A3 pseudogene	1.33E-11	-1.913
PSME4	Proteasome (prosome. macropain) activator subunit 4	2.29E-11	-1.899
CEP350	Centrosomal protein 350kDa	4.28E-11	-1.882
KDM4C	Lysine (K)-specific demethylase 4C	6.53E-11	-1.871
QSER1	Glutamine and serine rich 1	8.15E-11	-1.865
ZCCHC7	Zinc finger. CCHC domain containing 7	9.76E-11	-1.860
ZFAND6	Zinc finger. AN1-type domain 6	9.94E-11	-1.859
ARID1A	AT rich interactive domain 1A (SWI-like)	1.04E-10	-1.858
DENND1B	DENN/MADD domain containing 1B	1.15E-10	-1.855
RPS16P5	Ribosomal protein S16 pseudogene 5	2.27E-10	-1.837
UHMK1	U2AF homology motif (UHM) kinase 1	3.18E-10	-1.828
SEPT2	Septin 2	3.73E-10	-1.824
C21orf96	Chromosome 21 open reading frame 96	5.48E-10	-1.813
VPS13B	Vacuolar protein sorting 13 homolog B (yeast)	6.42E-10	-1.809
IL6ST	Interleukin 6 signal transducer (gp13. oncostatin M receptor)	1.38E-09	-1.788
SLC20A1	Solute carrier family 20 (phosphate transporter). member 1	1.41E-09	-1.787
UNQ6228	Hypothetical LOC100131541	1.59E-09	-1.784
SRGAP2	SLIT-ROBO Rho GTPase activating protein 2	1.64E-09	-1.783
SEPT9	Septin 9	1.78E-09	-1.781
CES1	Carboxylesterase 1 (monocyte/macrophage serine esterase 1)	2.28E-09	-1.774
SPEN	Spen homolog. transcriptional regulator (Drosophila)	2.76E-09	-1.768
CD44	CD44 molecule (Indian blood group)	3.42E-09	-1.762
BCL11A	B-cell CLL/lymphoma 11A (zinc finger protein)	4.33E-09	-1.756
FUBP3	Far upstream element (FUSE) binding protein 3	4.38E-09	-1.756
NFAT5	Nuclear factor of activated T-cells 5. tonicity-responsive	4.49E-09	-1.755
LYST	Lysosomal trafficking regulator	5E-09	-1.752
NIPBL	Nipped-B homolog (Drosophila)	5.03E-09	-1.752
PTPRC	Protein tyrosine phosphatase. receptor type. C	5.77E-09	-1.748
CCND2	Cyclin D2	6.84E-09	-1.743
IL6ST	Interleukin 6 signal transducer (gp13. oncostatin M receptor)	9.09E-09	-1.735
ARL17	ADP-ribosylation factor-like 17	9.4E-09	-1.734
IGHM	Immunoglobulin heavy constant mu	1.02E-08	-1.732

SFRS4	Splicing factor. arginine/serine-rich 4	2.31E-08	-1.709
DNAJC3	DnaJ (Hsp40) homolog. subfamily C. member 3	2.97E-08	-1.702
NBPF10	Neuroblastoma breakpoint family. member 10	3.07E-08	-1.701
FCRL5	Fc receptor-like 5	3.18E-08	-1.700
KLF12	Kruppel-like factor 12	3.41E-08	-1.698
MLL	Myeloid/lymphoid or mixed-lineage leukemia	3.63E-08	-1.696
AHNAK	AHNAK nucleoprotein	3.74E-08	-1.695

Table S11. Related to Figure 3. List of top significantly downregulated genes comparing PBMCs from high *vs*. low risk ABCD2 patients.

Table S12

Gene Symbols				
ABCB5	WASF2	RORA	IRF2BP2	SLC35F2
MALAT1	ID1	PTPRC	FHL5	FNBP4
HLA-DQA1	RAB38	ITPRIPL2	MUM1	DNAJC3
SMC3	FABP4	PRPF18	PCBP2	SRGAP2P1
CD36	HEATR7A	SNCA	SMG1	C5orf13
SLC16A6	MREG	UBN1	COL11A1	YME1L1
SLC16A10	SLC25A37	HOXB6	NR1D2	NRIP1
GREM1	TFRC	MNDA	KLF9	COL8A1
VAT1L	MERTK	SMPDL3A	NTM	LEFTY2
IL8	GALNT6	FAT3	TCF4	FNIP1
CDC42BPA	HAL	S100A8	RGS4	ZYG11B
STK4	NFIB	TPM1	PRKACB	SFRP4
ANKRD11	C6orf105	42063	EDIL3	PAPD4
SOD2	LILRA2	BCLAF1	EGFR	YPEL2
PHACTR2	FBP1	S100A12	C1S	CMTM4
PROK2	BAT2D1	MYO6	MPP7	ANKRD28
ANKRD12	DCUN1D1	CCL7	IGL@	ELN
GLUL	HSPA6	DKK3	COL12A1	SLMAP
TUBB1	C15orf48	FAM65B	DICER1	TNFRSF17
MMP8	FBXO32	PHF3	CRIM1	GBP3
CCDC88A	CPM	FRMD4A	SFRP1	MOXD1
UGCG	FN1	KYNU	SPON1	CBX5
HECTD1	TGS1	ZNF589	RABGAP1L	ASPN
RNF145	CYTL1	THRB	FSTL3	RDX
APOC1	BCAT1	ASPH	HAPLN1	RAVER2
BOD1L	ESF1	SDS	CNTN1	RWDD2B
IL8RB	CXCL16	PF4V1	HSPB6	C5orf28
IL1RN	LDB3	KCNJ15	CCDC8	SHC4
RIF1	NIPBL	MPHOSPH8	UFM1	NDUFA4L2
CLIP1	IGFBP1	MCTP1	NOV	IGHM
C10orf118	PCSK6	PTPRD	CLK4	DDX17
MYH10	SLC39A11	LPP	BACH2	POU2AF1
EML4	CD83	DDX60L	GAD1	DPT
HPGD	USP16	NEDD4L	ZNF621	NTRK2
SV2B	SFRS2IP	PARP15	MLL	PIK3R1
SLC11A1	RAB12	ATF3	PCMTD1	FAM76A
DOCK8	NPL	RGS7BP	ZNF207	LRRC16A
CXADR	COL4A3	C13orf18	MYOZ2	COCH
MRO	SCARB1	LGMN	LPHN3	CDC42
SCD	CTSLL3	ZNF281	DDR2	SPSB1
AQP9	TYMP	PLAUR	PDK4	SFRS11
S100P	SLC11A2	PDPN	SGEF	GLS
EMR2	BMP2K	HPSE	NKTR	NPR3
MLL3	SRRM2	PPP1R3C	MASP1	HLA-DQB1
FIGN	GK	RAB42	CCL19	BANF1
PALLD	ANXA1	KLHL6	FMO2	CPNE3
BCL2A1	ABCG1	IL18RAP	SLC16A7	TNRC6B
ACTA2	C6orf59	ACOT4	ARHGAP24	PDLIM7
HSP90B1	MCOLN3	ITGAX	SDK1	IGKV1OR15-118
ATP1A2	STEAP3	TBXAS1	FAM83D	CXCL9
CLDN23	SP3	BBX	MED13L	FAM20B
CYP4F3	PHLDB2	FLJ20674	HSP90AB1	PGGT1B
MARCO	NSMAF	SKAP2	BAG2	IGKV4-1
RGS16	CTSL1	GCHFR	ZEB1	IGJ
ADAM8	HAMP	CD53	UHMK1	IGKC

CCL18	POU4F1	CR1	FAM19A5	CXCL13
CP	ARGLU1	CEP290	ZNF652	
FOXC1	IGFBP5	NUCKS1	CALM1	
TOP1	CTSB	PAG1	TPM4	
HSPB8	MKNK1	CCL23	PLD5	
CYorf15B	PSD3	MAPK13	YWHAE	
APOE	TMCC3	EP400	C4orf18	
PKIB	OSCAR	MS4A14	LRRN1	
ALAS2	CCL4	SNX10	TMEM33	
UBASH3B	PGM2L1	CA2	CPXM2	
SPEN	ADAMTS5	SLA	KRT17	
MMP12	LILRB1	PTN	CLINT1	
RBM25	OSBPL8	PLD1	IGLJ3	
CADM3	CDO1	ABHD2	SLC7A8	
SLC2A5	LMO3	RSF1	FAM98A	
CALD1	THSD4	LIPG	CCND2	
CHD9	HS3ST2	ADAMTS8	AFF3	
KMO	TMEM163	RAP2A	C2orf67	
NUMA1	CNR1	MATR3	G3BP1	
CMTM2	MME	USP33	FAM46C	

Table S12. Related to Figure 4. List of significantly dysregulated genes in plaques common for CAR and ABCD2 comparisons from high *vs*. low risk patients.
